# *Fusobacterium nucleatum*-associated metabolites in disease pathogenesis: from cancer to immunotherapy

**DOI:** 10.3389/fmicb.2026.1996310

**Published:** 2026-10-26

**Authors:** Husni Farah, G. PadmaPriya, Samir Sahoo, Bahjat Alhasso, Shrey Kumar Srivastav, Anju Rani, Ritesh Singh, Ahmed Hassan Khdyr

**Affiliations:** 1 Faculty of Allied Medical Sciences, Hourani Center for Applied Scientific Research, Al-Ahliyya Amman University, Amman, Jordan; 2 Department of Chemistry and Biochemistry, School of Sciences, JAIN (Deemed to be University), Bangalore, India; 3 Department of General Medicine, IMS and SUM Hospital, Siksha ‘O’ Anusandhan (Deemed to be University), Bhubaneswar, India; 4 College of Pharmacy, Alnoor University, Nineveh, Iraq; 5 Department of General Medicine, Sharda University, Greater Noida, India; 6 Department of Nutrition and Dietetics, Chandigarh University, Mohali, India; 7 Centre for Research Impact and Outcome, Chitkara University, Rajpura, India; 8 Department of Pharmacy, College of Pharmacy, The Islamic University, Najaf, Iraq

**Keywords:** colorectal cancer, *Fusobacterium nucleatum*, immunomodulation, indole-derived compounds, microbial metabolites, short-chain fatty acids, succinate

## Abstract

*Fusobacterium*, especially *Fusobacterium nucleatum*, has emerged as a metabolically active pathobiont that can affect host physiology and disease pathogenesis. Through its ability to produce and regulate various metabolites, *Fusobacterium* can shape immune response, cellular metabolism, and disease progression. Short-chain fatty acids (SCFAs), being part of *Fusobacterium*-associated metabolic changes observed during disease, are involved in microbial cross-feeding and can affect neutrophil recruitment and inflammatory signaling through free fatty acid receptors. In the case of colorectal cancer (CRC), *Fusobacterium*-derived metabolites can impact tumor and immune cell phenotype via a number of mechanisms, including aryl hydrocarbon receptor (AhR) activation via indole derivatives, inflammatory and metabolic signaling via hydrogen sulfide, as well as an increase in Glucose transporter 1 (GLUT1) expression, lactate production, and histone lactylation via formate-related metabolites. Besides, *F. nucleatum* can reshape tumor-cell metabolism, including cholesterol biosynthesis through miR-130a-3p-mediated inhibition of AMP-activated protein kinase (AMPK) signaling, whereas butyrate has been reported to counteract this metabolic phenotype in experimental CRC models. In addition, *F. nucleatum*-associated succinate has been shown to suppress cGAS–Interferon beta (IFN-β) signaling, thereby reducing antitumor immune activation, impairing CD8^+^ T-cell trafficking, and promoting immune escape and resistance to anti-Programmed Cell Death Protein 1 (PD-1) therapy. The present review provides an overview of current understanding of the biological roles of *Fusobacterium*-derived metabolites, focusing on SCFAs, succinate, indole derivatives, hydrogen sulfide, and formate-related metabolites.

## 1 Introduction

*Fusobacterium nucleatum* is known as a causative agent of periodontal disease, colorectal cancer (CRC), intestinal inflammation, and other pathological states (Yang et al., 2025). Despite the numerous studies devoted to the adhesins, surface proteins, and other factors responsible for the virulence of *F. nucleatum*, the latest data show that metabolites of the bacterium also serve as a potential mechanism of pathogenicity. In this way, the bacterium can produce various metabolites, such as short-chain fatty acids (SCFAs), indole-derived compounds, succinate, hydrogen sulfide, formate, and others (Dahlstrand Rudin et al., 2021; Wang et al., 2023; Song et al., 2025). For example, SCFAs, primarily acetate and butyrate, produced by *F. nucleatum* may reach high concentrations capable of activating neutrophil responses via free fatty acid receptor 2 (FFAR2) activation, thus affecting inflammation and recruitment of inflammatory cells in periodontitis (Dahlstrand Rudin et al., 2021). Apart from local inflammation, *F. nucleatum*-associated metabolites can have an effect on tumor-cell metabolism, growth, invasiveness, immune responses, and antitumor therapy efficacy in the case of CRC. The findings identified *F. nucleatum*-associated indole metabolism and aryl hydrocarbon receptor (AhR)-dependent macrophage reprogramming, as well as changes in tumor immune cells and the progression of CRC (Song et al., 2025; Shimizu, 2026). Moreover, *F. nucleatum* metabolites can remodel host metabolism and epigenetic programs. For example, the *F. nucleatum*–formate–glucose transporter 1 (GLUT1)–lactate axis has been associated with altered glycolytic activity and increased histone lysine lactylation in CRC cells, together with changes in tumor-cell proliferation, migration, and invasiveness (Zhou et al., 2026). The effects of *Fusobacterium*-associated metabolites on immunology are also diverse. For example, in CRC, *F. nucleatum*-derived succinate affects the cGAS-mediated antitumor signaling and resistance to immune checkpoint blockade (ICB) (Wang X. et al., 2024). On the other hand, findings show that *F. nucleatum*-derived butyrate enhances anti-programmed cell death protein 1 (PD-1) responses in microsatellite-stable (MSS) CRC via increasing T-bet expression and decreasing PD-1 expression in CD8^+^ tumor-infiltrating lymphocytes (Wang X. et al., 2024). All these opposite effects show that the impact of *Fusobacterium* colonization cannot be predicted on the basis of its abundance or the presence of an individual metabolite, but depends on the interaction of bacterial localization, strain-specific metabolism, metabolite concentration, host genetics, immune status, tumor biology, and treatment. Previous reviews have comprehensively summarized the role of *F. nucleatum* in health and disease, including its contributions to inflammation, tumor development, immune regulation, and host–microbe interactions (Yang et al., 2025). Other recent reviews have further emphasized the capacity of *F. nucleatum* and related microbial factors to manipulate host immunity and influence tumor-associated immune responses and therapeutic outcomes (Li Q. et al., 2025). However, these reviews primarily consider *F. nucleatum* at the level of bacterial abundance, virulence factors, host–microbe interactions, or broader immune mechanisms. Our review adopts a complementary metabolite-centered framework, examining individual *Fusobacterium*-associated metabolites according to their demonstrated or proposed bacterial origin, experimental model, anatomical site, concentration or exposure context, cellular target, molecular pathway, disease phenotype, and treatment setting (Table 1). Finally, we assess the current evidence for diagnostic, biomarker, and therapeutic applications while identifying the limitations that currently prevent direct clinical translation. In this review, we describe current data about the metabolic products of *Fusobacterium*, their molecular interactions with host cells, contribution to inflammation and malignancy, use in diagnostics, and possible therapeutic targets.

**TABLE 1:** Function of *Fusobacterium*-derived metabolites in health and disease.

*Fusobacterium* spp.	Metabolite	Study setting	Health/disease	Function	References
*Fusobacterium* spp.	Butyrate	*In silico* comparative genomic analysis	Gut dysbiosis	*Fusobacterium* produces butyrate predominantly through amino acid–based pathways involving 4-aminobutyrate, glutarate, and lysine, in contrast to the pyruvate-dependent pathway used by many beneficial gut commensals. This metabolism is accompanied by ammonia production, suggesting that *Fusobacterium*-derived butyrate may have distinct ecological and host effects from commensal-derived butyrate.	Anand et al., 2016
*Fusobacterium nucleatum*	Butyrate (via butyryl-CoA)	Purified-enzyme biochemical assays investigating the *F. nucleatum* Ldh/ETF/Bcd system and genomic analysis of a 6-gene lactate-utilization cluster	Gut/oral microbial metabolism	*F. nucleatum* possesses a lactate-utilization pathway in which D-lactate is oxidized to pyruvate by lactate dehydrogenase (Ldh), with electrons transferred through electron transfer flavoprotein (ETF) to butyryl-CoA dehydrogenase (Bcd). This directly couples lactate oxidation to crotonyl-CoA reduction and butyryl-CoA formation, providing a route toward butyrate biosynthesis. The pathway may contribute to redox homeostasis during oxidative stress and indicates metabolic flexibility in *F. nucleatum.*	Do et al., 2025
*F. nucleatum*	Erucic acid	*In vitro* dynamic gastrointestinal pH model; *F. nucleatum* acid-survival assays; *F. nucleatum* FabM inhibition with cerulenin; mouse colonization of stomach and jejunum	Oral–gut axis/ gastrointestinal colonization	Membrane erucic acid enhances the ability of *F. nucleatum* to survive highly acidic gastric conditions (pH 1.5). Its synthesis is associated with the expression of the enoyl-CoA hydratase-related protein *F. nucleatum* FabM. Inhibition of FabM reduces membrane erucic acid levels and consequently decreases *F. nucleatum* survival and colonization of the stomach and jejunum, suggesting that this lipid-mediated acid resistance facilitates oral-to-gut translocation.	Li X. et al., 2025
*F. nucleatum*	Butanoate (butyrate)	Proteomic analysis of *F. nucleatum* in single-species culture and model oral microbial communities with *Porphyromonas gingivalis* and/or *Streptococcus gordonii*	Oral biofilm/periodontal microbial community	Integration of *F. nucleatum* into multispecies oral communities caused extensive metabolic remodeling, including reduced amino acid fermentation and a shift toward butanoate production as a metabolic end product. This metabolic adaptation may contribute to the ability of *F. nucleatum* to function as a metabolic bridge organism and support the development and maturation of periodontal biofilms.	Hendrickson et al., 2014
*F. nucleatum*	Butyrate	*In vitro* fermentation of *F. nucleatum* with dietary fibers (polydextrose and Fibersol-2); Caco-2 and NCM460 intestinal epithelial cells	Colorectal cancer (CRC)/dietary fiber–microbiota interaction	Dietary fibers markedly enhanced butyrate production by *F. nucleatum* (up to ∼206% with polydextrose and ∼153% with Fibersol-2). Fiber-conditioned *F. nucleatum* fermentation broth subsequently inhibited Caco-2 cell growth in a dose-dependent manner, suggesting that dietary fiber can shift *F. nucleatum*-associated activity toward an antitumor phenotype, potentially through increased butyrate production. The findings indicate that dietary composition may determine whether *F. nucleatum* exerts carcinogenic or potentially protective effects in the context of CRC.	Nawab et al., 2023
*F. nucleatum*	Butyrate	Microsatellite-stable (MSS) CRC patients; Fecal microbiota transplant (FMT) from *F. nucleatum*-high/low patients into germ-free mice; murine allografts and CD34^+^ humanized mice; intratumoral *F. nucleatum* administration and genetic knockout of a butyrate-producing gene	MSS CRC / anti-Programmed Cell Death Protein 1 (PD-1) immunotherapy	Intratumoral *F. nucleatum* produces abundant butyrate, which inhibits HDAC3/8 in CD8^+^ T cells, increases H3K27 acetylation at the *Tbx21* promoter, and induces *TBX21* expression. *TBX21* represses PD-1, thereby reducing CD8^+^ T-cell exhaustion and enhancing effector function. Consequently, *F. nucleatum*-derived butyrate sensitizes MSS CRC to anti-PD-1 therapy. Disruption of a butyrate-producing gene in *F. nucleatum* abolishes this immunotherapy-enhancing effect.	Wang X. et al., 2024
*F. nucleatum*	Butyrate, putrescine, and cadaverine	Multispecies oral biofilm models	Periodontal health and disease / oral biofilm dysbiosis	*F. nucleatum* metabolically integrates with commensals through amino-acid cross-feeding. *S. gordonii*-derived ornithine enhances putrescine production by *F. nucleatum*, while *V. parvula* increases lysine availability, promoting cadaverine production. Coculture also enhances butyrate production. *F. nucleatum*-derived polyamines create a putrescine-rich biofilm microenvironment that accelerates *P. gingivalis* biofilm maturation and dispersal, potentially promoting disease-associated oral biofilm development.	Sakanaka et al., 2022
*F. nucleatum (Fn7-1)*	Short-chain fatty acids (SCFAs)	Apc^Min/+ mice and gnotobiotic mice colonized with altered Schaedler’s flora; intestinal colonization model	Intestinal inflammation	*F. nucleatum* produces SCFAs in the intestinal tract that contribute to pro-inflammatory immune modulation through FFAR2-dependent signaling. SCFA–FFAR2 signaling promotes Th17-cell responses, increasing colonic IL-17A and IL-17F expression and associated IL-23p19 expression. This inflammatory environment occurs before tumor formation and potentiates intestinal tumorigenesis.	Brennan et al., 2021
*F. nucleatum*	Uric acid	DSS-induced ulcerative colitis (UC) mouse model with oral administration of *F. nucleatum*; 16S rRNA sequencing and LC-MS-based metabolomics	UC/ intestinal inflammation	*F. nucleatum* aggravated gut microbiota dysbiosis and metabolic disturbances, accompanied by a significant increase in uric acid, a metabolite associated with purine metabolism. The metabolic alteration occurred alongside increased intestinal inflammation, epithelial-cell apoptosis, and barrier dysfunction, suggesting that *F. nucleatum*-associated purine dysmetabolism may contribute to UC aggravation.	Lin et al., 2023
*F. nucleatum*	Propionic acid (propionate) and lactic acid (lactate)	CRC patient tissues, adjacent normal tissues, serum and feces; 16S rDNA microbiota profiling, ^1H-NMR metabolomics, targeted/untargeted mass spectrometry, and SW480 human CRC cells	CRC	*F. nucleatum* was associated with intestinal microbiota dysbiosis and altered metabolite profiles. Propionate concentrations were decreased, whereas lactate concentrations were increased in CRC tissues. *In vitro*, propionate promoted apoptosis, while lactate inhibited apoptosis of SW480 CRC cells. These metabolite alterations may therefore contribute to impaired CRC-cell apoptosis and tumor progression.	Yu et al., 2022
*F. nucleatum*	Butyrate	CRC patient tissues, CRC cell/tissue models, non-targeted metabolomics, and BALB/c mouse tumor models; PD-L1 blockade ± butyrate	CRC, MSI/dMMR and immunotherapy response	*F. nucleatum*-associated butyrate reduced MLH1 protein expression in CRC models, with pathway analyses and pharmacological inhibition implicating the autophagy–lysosome system. In CRC tissues, *F. nucleatum* abundance was associated with MSI and MLH1 deficiency. Combined butyrate and PD-L1 blockade produced greater tumor regression than either treatment alone in a subcutaneous mouse model. These findings support a potential *F. nucleatum*–butyrate–MLH1 pathway and possible immunotherapy-sensitizing effect, although direct causal conversion from MSS/pMMR to MSI/dMMR and the necessity of this pathway for treatment response require further validation.	Ding et al., 2025
*F. nucleatum*	SCFAs, primarily acetate and butyrate	*F. nucleatum* growth/metabolite analysis and human neutrophil chemotaxis and Ca^2 +^ signaling assays	Periodontal inflammation/periodontal disease	*F. nucleatum* releases high levels of acetate and butyrate as end-products of amino-acid fermentation. These SCFAs act as neutrophil chemoattractants through FFAR2 (GPR43) and induce cytosolic Ca^2 +^ signaling, promoting neutrophil recruitment to the periodontal pocket. This SCFA–FFAR2 axis may contribute to the inflammatory response associated with *F. nucleatum* colonization and potentially other *F. nucleatum-*associated diseases.	Dahlstrand Rudin et al., 2021
*F. nucleatum*	Indole and indole derivatives: indole, indole-3-acetic acid (IAA), indole-3-aldehyde, indole-3-carboxaldehyde, and indole-3-propionic acid (IPA)	Clinical *F. nucleatum* isolates from CRC, Crohn’s disease, healthy individuals, and oral sources; HPLC metabolite profiling, exogenous metabolite treatment, biofilm assays, virulence-gene expression analysis	CRC, Crohn’s disease, and healthy/oral states	CRC-associated *F. nucleatum* isolates produced significantly higher total indole levels than healthy isolates, and total indole production positively correlated with biofilm formation in CRC isolates. Exogenous indole derivatives differentially altered bacterial growth, biofilm formation, and virulence-associated gene expression, with CRC isolates showing distinctive responses and reduced carrying capacity following indole exposure. These findings suggest that indole metabolic profiles may contribute to niche-specific *F. nucleatum* virulence and biofilm-associated pathogenicity.	Scano et al., 2025
*F. nucleatum* and *Fusobacterium* spp.	Fusotrisindoline (FTIN), streptindole (STIN), and trisindoline (TIN)	*F. nucleatum* cultures, AHR reporter and CYP1A1 assays, CRC cell lines, ΔtnaA mutant, SNU-C4 xenograft model, and 30 paired human CRC/normal colon tissues	CRC	*F. nucleatum* produces these previously unrecognized indole-containing metabolites, which act as aryl hydrocarbon receptor (AHR) agonists. FTIN was the most potent ligand and activated the AHR–TERT axis, increasing TERT transcription and telomerase activity and thereby promoting CRC-cell proliferation, migration, invasion, and xenograft growth. Loss of tnaA abolished FTIN/STIN/TIN production and prevented AHR activation and *F. nucleatum-*mediated CRC growth. FTIN was also enriched in CRC tissues compared with matched normal colon tissues, suggesting potential value as a CRC-associated metabolite/biomarker.	Won et al., 2025
*F. nucleatum ATCC 25586*	Indole	*F. nucleatum* ATCC 25586; recombinant FN1943 enzyme assays, HPLC, gene-expression analysis, and planktonic/biofilm cultures exposed to L-tryptophan or exogenous indole	Oral microbial ecology/biofilm-associated periodontal disease	*F. nucleatum* converts L-tryptophan to indole through the tryptophanase-like FN1943 enzyme. Increasing L-tryptophan concentrations enhanced indole production and dose-dependently increased biofilm formation. Exogenous indole similarly promoted biofilm formation, indicating that indole functions as an extracellular signaling molecule regulating *F. nucleatum* biofilm development. High indole concentrations also suppressed fn1943 expression, suggesting a potential feedback-regulatory mechanism.	Sasaki-Imamura et al., 2010
*F. nucleatum*	Butyrate	CRC cells and mouse models treated with *F. nucleatum* ± butyrate; transcriptomics, miRNA analysis, TCGA analysis, and human fecal samples	CRC/hypercholesterolemia	*F. nucleatum* promotes host cholesterol biosynthesis by activating NF-κB and increasing miR-130a-3p, which suppresses AMPKα/β1, thereby activating SREBF2 and increasing cholesterol-biosynthetic genes including FDPS, FDFT1, and SQLE. Butyrate counteracted these effects, suppressing the *F. nucleatum*–miR-130a-3p–AMPK–SREBF2 pathway and reducing cholesterol accumulation. Increased fecal *F. nucleatum* and miR-130a-3p were associated with CRC and hypercholesterolemia.	Sun et al., 2025
*F. nucleatum*	Butyrate	Isolated *F. nucleatum* culture; capillary electrophoresis–time-of-flight mass spectrometry (CE-TOFMS) metabolomic profiling	Gastric cancer-associated microbial ecosystem	*F. nucleatum* predominantly produces butyrate through the acetyl-CoA pathway, demonstrating a distinct metabolic phenotype compared with *N. subflava* and *H. pylori*. This species-specific butyrate production may influence microbe–host and microbe–microbe interactions within the gastric environment and potentially contribute to gastric cancer-associated microbial metabolic alterations.	Niikura et al., 2025
*Fusobacterium varium*	Succinate	Fecal metagenomic analysis of UC patients and healthy controls; germ-free mice colonized with *F. varium*; DSS-induced colitis; bacterial culture supernatant fractionation and mass spectrometry; HL-60-derived neutrophils	UC/intestinal inflammation	*F. varium* is enriched in UC and produces succinate, which promotes neutrophil recruitment and exacerbates intestinal inflammation. Succinate activates SUCNR1 on neutrophils, triggering NF-κB signaling and inflammatory mediator production, including CXCL8. CXCR2 inhibition reduces neutrophil infiltration and attenuates colitis severity, supporting an *F. varium* → succinate → SUCNR1 → NF-κB → CXCL8/neutrophil recruitment → intestinal inflammation axis.	Wang et al., 2026
*F. nucleatum*	Succinic acid (succinate)	Metastatic CRC patients; fecal microbiota transfer and anti-PD-1 treatment in mouse models; *F. nucleatum* depletion with metronidazole	CRC/resistance to immunotherapy	*F. nucleatum*-derived succinate suppresses the cGAS–IFN-β pathway, reducing antitumor immune activation and limiting CD8^+^ T-cell trafficking into the tumor microenvironment. Consequently, elevated *F. nucleatum* and succinate promote resistance to anti-PD-1 immunotherapy. Metronidazole reduced *F. nucleatum* abundance and circulating succinate, restoring tumor sensitivity to immunotherapy.	Jiang et al., 2023

## 2 *Fusobacterium nucleatum*-derived metabolites and immunomodulation

*Fusobacterium nucleatum* is increasingly recognized not only as a microbial component associated with periodontal disease and CRC, but also as a source of bioactive metabolites capable of modifying host immune responses (Figure 1). The periodontal site colonization by bacteria triggers an intense inflammatory response, mainly mediated by the massive recruitment of neutrophils (Hajishengallis and Hajishengallis, 2014). The chemotaxis of neutrophils is primarily mediated by chemotactic G protein-coupled receptors (GPCRs) (Metzemaekers et al., 2020). Activation of GPCRs triggers various intracellular responses, including a rapid rise in cytosolic Ca2+ and chemotaxis, which is described as the directional movement of cells toward a rising agonist gradient (Metzemaekers et al., 2020). Several GPCRs activated by endogenous and bacterial agonists mediate the guidance of neutrophils from blood circulation toward the inflammatory sites (Abt et al., 2016). Endogenous chemoattractants, including Interleukin (IL)-8 and Leukotriene B4, usually produce a gradient that guides neutrophils from the bloodstream to the inflamed tissues (Abt et al., 2016). Thereafter, chemotactic signals produced by bacteria, such as formylated peptides, together with host-derived complement mediators such as C5a, contribute to further neutrophil migration toward the site of bacterial infection (Heit et al., 2002).

**FIGURE 1:**
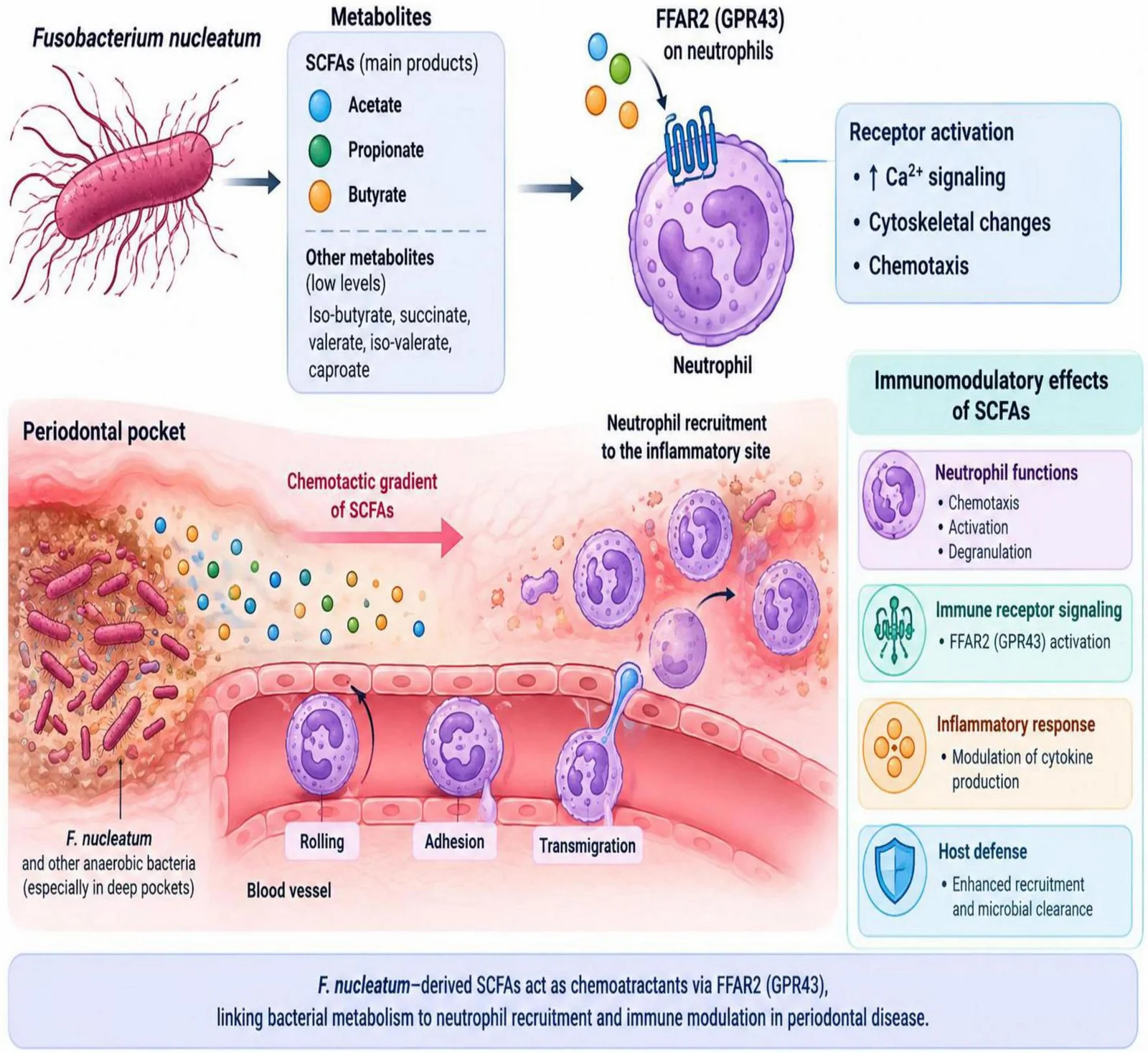
*Fusobacterium nucleatum*-derived metabolites and immunomodulation. *Fusobacterium nucleatum*, an anaerobic, proteolytic, amino acid–fermenting bacterium, produces substantial amounts of short-chain fatty acids (SCFAs), particularly acetate, propionate, and butyrate, through amino acid degradation. Acetate is detected at millimolar concentrations, whereas propionate and butyrate occur at high micromolar concentrations in *F. nucleatum* culture supernatants; iso-butyrate, succinate, valerate, iso-valerate, and caproate are present at comparatively lower concentrations. SCFAs activate free fatty acid receptor 2 (FFAR2/GPR43), a G-protein-coupled receptor abundantly expressed on neutrophils, triggering intracellular Ca^2 +^ signaling and promoting chemotaxis. In the periodontal pocket, SCFAs generated by *F. nucleatum*, together with metabolites produced by other bacteria residing in deep periodontal sites, can establish a chemotactic gradient that facilitates neutrophil recruitment and contributes to local inflammatory responses. The figure summarizes the proposed link between *F. nucleatum* amino acid fermentation, SCFA production, FFAR2-dependent signaling, and neutrophil influx, highlighting bacterial metabolites as mediators of host–microbe immune communication.

SCFAs, including acetate, propionate, and butyrate, are among the significant groups of bacteria-derived neutrophil chemoattractants. These metabolites are mainly produced by anaerobic gut bacteria through carbohydrate fermentation and have multiple regulatory roles in metabolic and immune responses (Alvarez-Curto and Milligan, 2016). Each of the SCFAs shows a specific affinity for FFAR2, also known as GPR43, which is a chemoattractant GPCR abundantly expressed on neutrophils (Nilsson et al., 2003). Although the formation of SCFAs through dietary fiber fermentation by gut bacteria has been largely studied, bacterial amino acid fermentation is another potential way to produce high amounts of these metabolites. For example, *F. nucleatum* generates high amounts of acetate, propionate, and butyrate (Barker et al., 1982). Although this bacterium ferments glucose only in relatively low amounts, most SCFAs secreted by *F. nucleatum* are produced due to amino acid degradation (Bolstad et al., 1996). Since the production of SCFAs can be considered plausible small hydrophilic chemotactic factors in the *F. nucleatum* culture supernatants, Dahlstrand Rudin et al. (2021) determined the concentrations of these metabolites using liquid chromatography combined with tandem mass spectrometry. The *F. nucleatum* culture supernatants contain high concentrations of acetate (millimolar concentration) as well as propionate and butyrate (high micromolar concentrations). At the same time, the concentrations of iso-butyrate, succinate, valerate, iso-valerate, and caproate were low. To test if SCFAs act through FFAR2 as chemotactic factors of *F. nucleatum* supernatants, a specific FFAR2 antagonist, CATPB, was used to inhibit the Ca2+ responses triggered by *F. nucleatum* (Dahlstrand Rudin et al., 2021). Treatment of neutrophils with *F. nucleatum* FT fractions and CATPB nearly completely inhibited Ca2+ responses. Although CATPB was unable to fully block the Ca2+ responses evoked by *F. nucleatum* unseparated supernatants, these responses were significantly reduced. These results indicate that FFAR2 plays a major role in neutrophil responses to *F. nucleatum* supernatants. In addition, Dahlstrand Rudin et al. (2021) demonstrated that intense amino acid fermentation performed by *F. nucleatum* can produce high amounts of SCFAs. Therefore, SCFAs generated by *F. nucleatum* and also by other bacteria inhabiting deep periodontal pockets are likely to contribute significantly to neutrophil influx characteristic of periodontal disease (Hajishengallis and Hajishengallis, 2014). Overall, the presented results further support the idea that the mechanism of SCFA-FFAR2 signaling is a significant process that mediates neutrophil recruitment in the periodontal pocket. Besides periodontal disease, this mechanism might also be involved in pathological processes associated with colonization by *F. nucleatum* or other anaerobic bacteria that use proteins as their main energy source.

Recent evidence has further emphasized the broader translational relevance of SCFAs, including their potential influence on host metabolism, drug-metabolism processes, inflammatory and metabolic disorders, and microbiome-targeted therapeutic strategies (Sankarganesh et al., 2025). Importantly, SCFA production and biological activity are highly context-dependent and can vary according to dietary substrate availability, gut microbial composition, host genetics, anatomical location, and disease state. Thus, although SCFAs are frequently discussed as beneficial microbial metabolites, their effects depend on the individual SCFA, its concentration, the cellular target, and the surrounding microenvironment. This broader physiological framework is particularly relevant when considering SCFAs produced by disease-associated bacteria such as *F. nucleatum*, whose metabolic activity may differ substantially from that of canonical commensal SCFA-producing bacteria. Indeed, acetate and propionate were demonstrated to induce neutrophil chemotaxis *in vitro* at micromolar to millimolar concentrations (Dahlstrand Rudin et al., 2021). In accordance with these data, Vinolo et al. (2009) demonstrated that millimolar concentrations of acetate, propionate, and butyrate serve as neutrophil chemoattractants *in vivo* using an air pouch rat model. In light of this, Dahlstrand Rudin et al. (2021) by liquid chromatography with tandem mass spectrometric analysis demonstrated that *F. nucleatum* culture supernatants contain millimolar concentrations of acetate, whereas propionate and butyrate are found in supernatants at high micromolar concentrations. Isobutyrate, succinate, valerate, iso-valerate, and caproate were found in relatively lower concentrations. Additional arguments in favor of the collective contribution of these metabolites follow from previous findings that combined SCFAs, including acetate, propionate, butyrate, iso-butyrate, succinate, iso-valerate, and caproate, induce FFAR2-dependent Ca2+ signaling and neutrophil chemotaxis *in vitro* (Dahlstrand Rudin et al., 2020). The concentration profile of SCFAs measured in *F. nucleatum* culture supernatants, together with the detection of SCFAs in gingival crevicular fluid (GCF) from patients with periodontitis, provides evidence that these metabolites may be present within the periodontal inflammatory environment. However, concentrations measured in bacterial culture supernatants or used in functional assays should not be assumed to represent the free concentrations experienced by neutrophils *in vivo*. Differences in local pH, dilution, metabolite transport and uptake, protein binding, oxygen availability, and spatial localization within the periodontal pocket may substantially influence cellular exposure. Therefore, the observed FFAR2-dependent neutrophil responses support a potential mechanistic role for SCFAs but do not establish that the concentrations effective *in vitro* are quantitatively equivalent to those at the host-cell surface in the periodontal pocket. Some individual SCFAs have been shown to induce FFAR2-dependent Ca2+ signaling and neutrophil chemotaxis (Dahlstrand Rudin et al., 2021). Additional evidence of the association between FFAR2-dependent neutrophil recruitment and SCFAs generated by bacteria fermenting fiber comes from murine colitis models (Sina et al., 2009). Expanding these findings to an oral pathogen, the current findings indicate that intense amino acid fermentation by *F. nucleatum* leads to the formation of SCFAs in high concentrations. Therefore, SCFAs formed by *F. nucleatum*, together with SCFAs formed by other bacteria that inhabit the deep periodontal pocket, can contribute to neutrophil influx characterizing periodontal diseases (Hajishengallis and Hajishengallis, 2014). This mechanism can be applied to pathologies not only in periodontal lesions. Plaque-induced inflammation can also appear in the soft tissues surrounding dental implants in the form of mucositis, and it can progress to bone loss, which is called peri-implantitis (Berglundh et al., 2018). Compared to periodontitis lesions, peri-implant lesions demonstrate a higher neutrophil infiltration (Berglundh et al., 2011). Since *F. nucleatum (subsp. nucleatum*) was recently identified as the bacterial species with particular relevance during the transition from mucositis to the more aggressive stage of peri-implantitis, this SCFA-FFAR2 axis can be involved in the pathogenesis of peri-implant diseases (Ghensi et al., 2020).

Although the majority of mechanistic evidence discussed in this review concerns *F. nucleatum*, recent work on other *Fusobacterium* species provides complementary evidence that metabolite-mediated host interactions may also contribute to disease. In particular, Wang et al. (2026) identified *Fusobacterium varium*-derived succinate as an inflammatory metabolite in ulcerative colitis (UC). In this study, *F. varium* colonization exacerbated dextran sodium sulfate (DSS)-induced colitis, while succinate activated SUCNR1-dependent nuclear factor kappa-light-chain-enhancer of activated B cell (NF-κB) signaling in neutrophils and promoted inflammatory mediator production. Importantly, this finding represents evidence for a *F. varium*–succinate pathway and should not be interpreted as direct evidence for *F. nucleatum* biology. Whether *F. nucleatum* produces succinate at comparable levels or engages the same SUCNR1-dependent pathway in intestinal inflammation remains to be established. Along with known virulence-associated determinants of *F. varium*, such as FadA adhesins and secretion systems, Wang et al. (2026) showed secreted metabolites as another group of host-interacting factors that are able to stimulate NF-κB signaling and drive mucosal inflammation. This idea is in line with numerous data about pro-inflammatory properties of microbial metabolites, including cadaverine, bile acid derivatives, and indolimine (Wang et al., 2026). Together, this gives rise to an extended paradigm of the interaction between host and microbiota, where gut bacteria affect host immunity not only via microbial structural determinants and classical virulence factors but also via chemical compounds that have a biologically active nature. However, the inflammatory activity of *F. varium* culture supernatant might not be exclusively driven by succinate. Some other heat-stable, low molecular weight metabolites present in the culture supernatant could contribute independently or synergistically to the observed activity. In Wang et al. (2026) study, an activity-guided fractionation of *F. varium* culture supernatant identified succinate as one of the main NF-κB activators. Despite the identification of kynurenic acid, its relatively weak and non-dose-dependent activity suggests that this compound is not a major NF-κB activator in this system. Thus, succinate can be considered a major bioactive metabolite of *F. varium*, while the presence of additional cooperating metabolites remains possible. Despite the historical view of succinate as a simple intermediate of the tricarboxylic acid (TCA) cycle, it is now clear that succinate is an important immunometabolite which can accumulate during inflammation and regulate immune response via SUCNR1. Wang et al. (2026) results expand this idea by showing that succinate aggravates DSS-induced colitis and is associated with neutrophil recruitment, thus implicating the excess of succinate in neutrophil-driven inflammation. It is important to note that succinate-SUCNR1 signaling is not generally pro-inflammatory and exerts context-dependent immunoregulatory effects. Thus, the biological effect of succinate accumulation depends on cell population and microenvironment. Further investigation of spatial distribution, sources, and sensing mechanisms of succinate in the intestinal mucosa would be necessary to understand the role of succinate-SUCNR1 signaling in UC pathogenesis and determine whether this pathway is a potential target for therapy.

By using murine gnotobiotic models, Brennan et al. (2021) studied the immunological effects of *F. nucleatum* colonization and observed that this bacterium promoted colonic *Il17a* gene expression and induced both expansion of intestinal T helper 17 (Th17) cells and IL-17 family cytokine production. This is reminiscent of the immunological phenotypes associated with ETBF-driven tumorigenesis and suggests a possible convergent mechanism between the two CRC-associated microbes. Furthermore, their data indicate that SCFAs, metabolites produced by *F. nucleatum* in the gut, may mediate the immunomodulatory activity of this oncomicrobe. Specifically, Brennan et al. (2021) demonstrated that *F. nucleatum*, another oncomicrobe associated with CRC, can modulate intestinal Th17 cell numbers and the expression of IL-17 family cytokines. Under homeostatic conditions, the effect of *F. nucleatum* strain Fn7-1 on Th17 cells was modest. Nonetheless, this effect could potentially become more prominent in the presence of secondary disturbances, including chronic inflammation or antibiotic therapy. In addition, although enhanced IL-17 expression was observed before tumor development in the neonatal colonization model, the contribution of IL-17 signaling to the formation and programming of the tumor microenvironment in *F. nucleatum*-associated CRC requires further study. Brennan et al. (2021) also reported that colonization with Fn7-1 increased intestinal SCFA levels. Importantly, the expression of genes encoding FFAR2 and NIACR1, receptors for SCFAs, was upregulated in human CRC samples infected with *F. nucleatum*, suggesting that these tumors could be susceptible to SCFA-mediated immunomodulation.

The failure to observe an increase in Th17 cell numbers in Fn7-1-colonized ASF *Ffar2-/-* mice may be due to differences in microbiota composition in this strain rather than the lack of SCFA sensing through FFAR2. Whether activation of FFAR2 alone is sufficient to generate this effect also remains unclear. If FFAR2 activation is insufficient, another signal specific to fusobacteria may be required to cooperate with this more general SCFA-mediated immunomodulatory pathway. Therefore, the contribution and mechanistic role of FFAR2 in the *F. nucleatum*-induced Th17 response remain to be determined. The expression of Ffar2 in colonic Th17 cells further suggests that this effect may occur through a Th17 cell-intrinsic pathway; however, the downstream signaling pathways through which FFAR2 regulates Th17 cell recruitment and differentiation remain to be identified. Neutrophils are an important source of IL-23, a cytokine that promotes Th17 cell recruitment (Kvedaraite et al., 2016). Consistently, upregulation of IL-23 expression in the colonic LP of *F. nucleatum*-colonized mice provides a potential framework through which colonization and SCFA sensing may influence the Th17 response. Data from genetic mouse models further support a potential protective role of SCFA signaling in CRC. Specifically, deficiency in Ffar2 or Niacr1 in *ApcMin/+* mice leads to increased intestinal tumor burden, demonstrating the capacity of SCFA signaling through these receptors to inhibit tumor development (Brennan et al., 2021). However, the biological effects of individual SCFAs are not uniformly protective. For instance, butyrate promotes colonic epithelial proliferation and causes tumorigenesis in *Apc* ^*Min*/+^ and *Msh2*^–/–^ mice (Belcheva et al., 2014). These apparently contradictory findings demonstrate the context dependency of metabolite-mediated immune and epithelial responses. Factors such as genetic background, environmental and microbial stimuli, the availability of specific SCFAs within particular anatomical niches, and the ability of cells to access these metabolites may all influence the final outcome. In the particular context of *F. nucleatum* colonization, SCFA signaling could contribute to alterations in the intestinal Th17 response and the formation of an immune microenvironment favorable for tumor development. Although the contribution of FFAR2 and other SCFA-sensing pathways requires further clarification, this experimental system provides an opportunity to further explore how *F. nucleatum*-derived metabolites influence antitumor immunity and the tumor microenvironment in CRC.

Recent studies have demonstrated that the impact of *F. nucleatum* on CRC involves not only bacteria-host interactions but also production of bioactive metabolites able to modulate the tumor immune microenvironment. In particular, indole derivatives that could act as ligands for the AhR, the ligand-activated transcription factor integrating microbial, dietary, and endogenous inputs in the regulation of immune-cell function, attract attention (Song et al., 2025). Song et al. (2025) discussion provides evidence that 3-indolepropionic acid (IPA) produced by *F. nucleatum* is an important metabolic mediator connecting bacterial colonization with immunosuppressive reprogramming of macrophages in CRC. Macrophages are highly plastic immune cells residing in the CRC tumor microenvironment (TME), and the functional phenotype of macrophages has a great impact on tumor development. Despite the simplification implied in the classic M1/M2 classification of macrophages, tumor-associated macrophages with an M2-like or immunoregulatory phenotype usually favor tumor growth, angiogenesis, tissue remodeling, and inhibition of antitumor immunity (Song et al., 2025). As shown in Song et al. (2025) study, the treatment of macrophages with *F. nucleatum* culture supernatant results in induction of CD206 expression and upregulation of CD163, IL-10, transforming growth factor-beta (TGFβ), and vascular endothelial growth factor (VEGF). The latter finding indicates that a metabolic mediator produced by *F. nucleatum* is able to recapitulate a considerable part of the immunomodulatory potential of the bacterial secretome. Importantly, the identification of IPA as an immunoregulatory metabolite produced by *F. nucleatum* is quite relevant since the indole metabolites constitute an important intersection point of microbial metabolism and host immune signaling (Song et al., 2025). Intestinal bacteria metabolize tryptophan into different indoles, activating AhR in epithelial cells and immune populations. Activation of AhR may control transcriptional programs related to macrophage differentiation, inflammation, cytokine production, and tissue homeostasis. Thus, the conversion of tryptophan by bacteria into active AhR ligands is the mechanism through which the alterations in the intestinal microbiota can lead to changes in the local or systemic immune function. As follows from the findings provided in the study under discussion, IPA–AhR signaling favors the formation of immunosuppressive macrophages in the CRC TME. Mechanistically, IPA activates AhR in macrophages and leads to its translocation to the nucleus, followed by the initiation of the transcriptional program typical for M2-like macrophage polarization (Song et al., 2025). M2-like polarized macrophages can promote CRC progression through the combination of several interconnected mechanisms. An increase in IL-10 and TGF-β expression leads to the inhibition of effector immune responses, while VEGF production is beneficial for tumor-associated angiogenesis (Song et al., 2025). At the same time, tumor-promoting macrophages can alter the process of extracellular matrix remodeling and provide growth and survival signals required for tumor cell migration and invasion. Thus, IPA-induced macrophage reprogramming establishes a positive feedback loop in which microbial metabolism results in immune suppression and further permissiveness to tumor development. One of the particularly interesting observations is the decrease in CD8^+^ T cell abundance after treatment with *F. nucleatum* culture supernatant or IPA. Cytotoxic CD8^+^ T cells represent a key component of adaptive antitumor immunity, and the exclusion, dysfunction, or depletion of CD8^+^ T cells are frequently connected with impaired tumor immune surveillance (Song et al., 2025). The findings demonstrate that the effects of *F. nucleatum*-derived metabolites extend beyond macrophage polarization, affecting the TME immune cell composition. However, one should be careful about the interpretation of the findings: the decrease in CD8^+^ T cells does not prove either that IPA acts directly on T cells or that the observed effect is secondary to macrophage reprogramming. In the latter case, the findings are consistent with the previously suggested model of IPA action since AhR activation induced by IPA was shown in macrophages, and the effects were partially inhibited by AhR antagonist CH223191 (Song et al., 2025). Inhibition of AhR by CH223191 is quite an important part of the study because it demonstrates the involvement of AhR in IPA-induced M2-like polarization and tumor growth. This observation puts the findings into the context of the wider concept according to which different indole metabolites produced by bacteria can have distinct immunological effects depending on their affinity, concentrations, stability, target cells, and local microenvironment. As a result, the effects of bacterial tryptophan metabolism on the immune system are unlikely to be determined by the presence/absence of indole metabolites only but depend on the quantitative and qualitative composition of the pool of metabolites generated by microbiota. The findings extend the concept of *F. nucleatum* as an immunomodulatory microorganism in CRC developed in previous studies. The role of this bacterium in CRC was discussed primarily in the context of direct mechanisms of bacterial activity, including interactions with epithelial and immune-cell receptors, regulation of inflammatory signaling, and alteration of immune-cell function (Song et al., 2025). Song et al. (2025) provided evidence that IPA-associated AHR activation can contribute to immunosuppressive macrophage reprogramming in CRC. Their findings showed that *F. nucleatum* culture supernatant and exogenous IPA activated AHR in macrophages, increased M2-associated markers, and were associated with reduced CD8+ T-cell infiltration and enhanced tumor growth in preclinical models. However, these findings should be interpreted within the experimental context of that study. In particular, the demonstration of IPA-associated activity does not establish IPA as a universal metabolite produced by all *F. nucleatum* strains. The contrasting observation that IPA was not detected in the *F. nucleatum* cultures examined by Won et al. (2025), together with their identification of FTIN, STIN, and TIN as AHR agonists, indicates that the AHR-active metabolite profile of *F. nucleatum* is dependent on bacterial strain, substrate availability, and culture conditions. Therefore, IPA should be considered a context-dependent candidate mediator of *F. nucleatum*–AHR signaling rather than a species-wide metabolic signature. At present, neither IPA nor fusotrisindoline (FTIN)/streptindole (STIN)/trisindoline (TIN) should be considered clinically validated biomarkers of CRC progression or treatment response, because their reported effects derive predominantly from experimental models and, for FTIN/STIN/TIN, from recently emerging evidence requiring independent validation. Indeed, the bacterial secretome contains various metabolites, and IPA might work in combination with other compounds secreted by *F. nucleatum* to affect macrophage functionality. As such, the biological phenotype associated with IPA in the tumor microenvironment could result from the activity of an entire metabolic signature rather than one specific metabolite. In the future, functional studies need to define whether IPA works in synergy with or independently of other metabolites produced by *F. nucleatum*. The therapeutic potential of these results includes targeting the axis of *F. nucleatum*–tryptophan metabolism–IPA–AhR–macrophage reprogramming. These findings provide a preclinical rationale for investigating approaches that selectively modulate bacterial IPA production, tryptophan metabolism, or *F. nucleatum*-associated microbial pathways. However, the therapeutic efficacy, safety, and feasibility of these strategies in patients with CRC remain unknown. However, the global inhibition of AhR can bring undesirable consequences since AhR plays an important role in the mucosal immune response, maintenance of barrier function, and immune homeostasis. Therefore, approaches that would specifically target tumor-associated microbial metabolism or locally affect AhR activation are preferable. Taken together, current evidence supports a role for *F. nucleatum*-associated metabolites in AHR-mediated regulation of CRC progression, but the responsible metabolite appears to be context-dependent. IPA has been reported to activate AHR in macrophages and promote an immunosuppressive phenotype in CRC models, whereas FTIN, STIN, and TIN have been independently identified as AHR agonists, with FTIN producing a strong AHR–Telomerase Reverse Transcriptase (TERT) response in susceptible CRC cell lines. The failure to detect IPA under the culture conditions used by Won et al. (2025) indicates that production of individual AHR-active metabolites is not uniform and may depend on strain, substrate, and environmental conditions. Thus, the *F. nucleatum*–AHR axis should be viewed as a metabolically heterogeneous pathway rather than being attributed to IPA alone.

## 3 *Fusobacterium nucleatum*-derived metabolites and host cholesterol biosynthesis

Beyond its effects on inflammatory and immune pathways, *F. nucleatum* has been linked to alterations in host cholesterol metabolism. Rather than referring to a “cholesterol-synthesizing microbiome,” the available evidence supports a model in which *F. nucleatum* influences cholesterol biosynthesis and regulatory pathways in host cells. Sun et al. (2025) results revealed *F. nucleatum* as an overlooked cholesterol-synthesizing microbiome, affecting de novo cholesterol synthesis in the host intestine and possibly causing disturbances in systemic cholesterol homeostasis. At the molecular level, they demonstrated that *F. nucleatum* stimulated cholesterol biosynthesis in CRC through up-regulation of miR-130a-3p, which inhibited AMPK signaling. On the other hand, butyrate displayed a contrasting effect, indicating the possible use of SCFA in reducing the negative impact of *F. nucleatum* on the host’s metabolism. Sun et al. (2025) results are the first ones showing that a gut bacterium is capable of regulating host endogenous cholesterol biosynthesis, especially in CRC, expanding the knowledge about microbiota-induced regulation of cholesterol metabolism. The described mechanisms could help us understand metabolic adaptations occurring in CRC patients and suggest new dietary interventions, including the use of butyrate, in overcoming metabolic consequences caused by pathogenic gut bacteria. It is already known that *F. nucleatum* infection leads to the stimulation of hepatic lipogenesis and aggravation of atherosclerosis in mice with a genetic predisposition under a high-fat and high-cholesterol diet (Zhou et al., 2023). Since the liver is one of the major organs responsible for cholesterol biosynthesis and is extensively connected with the gut via the gut-liver axis, excessive presence of *F. nucleatum* in the intestine could somehow affect hepatic cholesterol metabolism. However, in a 12-week *F. nucleatum* gavage-induced mouse model, there were no significant changes in the concentration of cholesterol in the liver (Sun et al., 2025). This lack of phenotypic effect on the liver could be explained by insufficient length of the treatment time. Thus, longer periods of time or higher doses of bacteria will need to be applied to investigate the ability of intestinal *F. nucleatum* to affect hepatic cholesterol biosynthesis and to reveal its mechanisms. Nevertheless, the correlation between the high abundance of *F. nucleatum* in feces and the dysregulation of cholesterol metabolism means that patients with high *F. nucleatum* levels in feces or periodontal infection by *F. nucleatum* should pay special attention to the status of their blood cholesterol levels (Sun et al., 2025). *F. nucleatum* contributes to CRC development via several molecular mechanisms, including the activation of oncogenic NF-κB and E-cadherin/β-catenin signaling pathways (Sun et al., 2025). In addition, *F. nucleatum* colonization is accompanied by considerable host gene regulation, including upregulation of the oncogenic miR-21 and long non-coding RNA ENO1-IT1 and downregulation of miR-18a and miR-4802. Sun et al. (2025) demonstrated that *F. nucleatum* stimulates the expression of miR-130a-3p through NF-κB activation. Several mechanisms have previously been reported to contribute to *F. nucleatum*-induced NF-κB activation, including engagement of TLR4 by its lipopolysaccharides, interaction with CD147 through the adhesin RadD, and activation of the pattern-recognition receptor ALPK1 by the bacterial metabolite ADP-heptose. However, additional studies are required to determine the precise signaling events linking *F. nucleatum*-induced NF-κB activation to subsequent stimulation of cholesterol biosynthesis (Sun et al., 2025).

The induced miR-130a-3p inhibited the expression of PRKAA1 (AMPKα) and PRKAB1 (AMPKβ1), thereby reducing pAMPKα levels and AMPK activity. In mouse models with relatively longer treatment periods, this reduction in pAMPKα was more pronounced than that observed in cells. Further inhibition of AMPK led to activation of SREBF2 and increased expression of genes involved in cholesterol biosynthesis. These findings are consistent with previous studies reporting NF-κB-mediated induction of miR-130a-3p in cervical cancer cells and miR-130a-3p-mediated inhibition of AMPKα and AMPKβ expression, accompanied by increased lipid accumulation in orbital fibroblasts (Zhang et al., 2014; Hammond et al., 2021). Furthermore, the interaction between AMPK signaling, SREBF2 activation, and cholesterol biosynthesis has been established in hepatic and auditory cells (Tang et al., 2016; Wang et al., 2019). In line with these observations, Sun et al. (2025) found that AMPK inhibition decreased INSIG2 expression and stimulated the expression of SREBF2 target genes involved in cholesterol biosynthesis in colon cancer cells, whereas pharmacological activation of AMPK markedly suppressed the *F. nucleatum*-induced expression of cholesterol biosynthesis genes. Collectively, these findings support a model in which *F. nucleatum* promotes cholesterol biosynthesis through AMPK suppression and SREBF2 activation.

Further evidence for the clinical relevance of this pathway comes from the observation that CRC patients exhibited increased levels of plasma exosomal miR-130a. Consistent with these findings, Sun et al. (2025) reported elevated fecal miR-130a-3p levels in patients with CRC and advanced adenomas, which were associated with increased fecal *F. nucleatum* abundance. The study also identified an association between high fecal *F. nucleatum* abundance and hypercholesterolemia. Notably, this association was observed despite statin treatment in all patients with hypercholesterolemia at the time of stool collection, consistent with earlier observations that *F. nucleatum* is relatively insensitive to the direct antibacterial effects of statins. Although univariate and multivariate logistic regression analyses identified high *F. nucleatum* abundance as the main factor associated with hypercholesterolemia, this association did not reach statistical significance. The interpretation of these findings is further limited by the retrospective nature of the cohort, which prevents adequate control for potential confounders, including dietary patterns and other lipid-lowering interventions that were not documented. Similarly, the TCGA cohort does not provide information regarding non-cancer medications, including cholesterol-lowering agents. Therefore, prospective studies incorporating comprehensive medication, dietary, and metabolic information are required to validate the relationship between *F. nucleatum* abundance, cholesterol biosynthesis gene expression, and hypercholesterolemia. Beyond identifying a potential role for *F. nucleatum* in host cholesterol biosynthesis, the study suggests that butyrate and butyrate-producing bacteria may counteract these adverse metabolic effects, providing a potential strategy for mitigating *F. nucleatum*-associated hypercholesterolemia. Butyrate is a major SCFA produced by beneficial gut bacteria and exerts diverse biological effects, including serving as an energy source for intestinal cells and contributing to antioxidant, anti-inflammatory, and intestinal barrier-preserving activities. Overall, current evidence supports a model in which *F. nucleatum* can modulate host cholesterol biosynthesis and related metabolic signaling, particularly in CRC, while the precise relationship among AMPK, INSIG proteins, SREBF2, and cholesterol metabolism depends on the tissue and experimental context. Further comparative studies are needed to determine whether these mechanisms operate similarly in hepatic and colorectal tissues and whether the observed metabolic changes are consistently associated with disease progression in patients.

## 4 Function and mechanism of *Fusobacterium nucleatum*-derived metabolites in diseases

The *F. nucleatum* strain is currently regarded as an organism that has high metabolic activity among disease-eliciting microbes, whereby the metabolites of this organism play a role in the interactions between the microbiota and the tissues. This is through the production and modification of SCFAs, products of amino acids, sulfur metabolites, polyamines, and other acids; as such, *F. nucleatum* can alter the functions of the epithelial barrier, metabolism, oxidative stress, immune system response, and inflammation. This is mainly applicable in diseases such as periodontal disease and CRC.

### 4.1 Cancer

Of the many intratumoral bacteria studied with respect to CRC, *F. nucleatum* stands out for having the most comprehensive body of literature (Wang S. et al., 2021). Profiling the composition of the microbiota in CRC tissues and matched adjacent normal mucosa, as well as comparisons of stool samples from CRC patients with those from healthy subjects, consistently indicate a high abundance of *F. nucleatum* in CRC (Won et al., 2025). Mechanistic studies showed that *F. nucleatum* makes use of multiple macromolecular adhesins, including CbpF, FadA, Fap2, and RadD, to colonize CRC tissues and influence tumor-associated pathophysiology and immunity. Recently, the role of *F. nucleatum* was expanded further by showing that *F. nucleatum* not only utilizes macromolecular adhesins but also produces biologically active metabolites that affect the progression of CRC, including activation of oncogenic signaling, tumor cell proliferation and metastasis, and chemoresistance (Won et al., 2025). Some of the metabolites produced by *F. nucleatum* include DL-homocystine and allantoic acid, which were reported to cause DNA damage in the SW480 human CRC cell line and a mouse model (Figure 2; Xu et al., 2024). Additionally, ADP-heptose and butyrate act together to stimulate NF-κB signaling, leading to promotion of proliferation and 5-fluorouracil resistance in HT-29 human CRC cells (Martin-Gallausiaux et al., 2024). Fn-produced succinic acid has been shown to enhance resistance to ICB in MC38 and CT26 mouse CRC cells (Jiang et al., 2023). Further, hydrogen sulfide produced by *F. nucleatum* via metabolism of L-cysteine increases the migratory capacity of HCT116 and DLD-1 cells, but not RKO cells (Wang et al., 2023). Lastly, two other metabolites have been shown to affect CRC via AHR signaling. The formate-mediated increase in AHR activity leads to increased cancer stemness in a mouse xenograft model of CRC involving HT-29 cells (Ternes et al., 2022). On the other hand, IPA has been reported as an AHR-activating metabolite associated with *F. nucleatum* culture supernatant and was shown to activate AHR in macrophages and promote CRC-related phenotypes in the experimental models examined by Song et al. (2025). However, IPA production should not be considered a universal feature of *F. nucleatum*, because its detection appears to depend on bacterial strain and experimental culture conditions (Song et al., 2025). Thus, *F. nucleatum* not only uses macromolecular adhesins but also produces small-molecule metabolites that influence CRC progression.

**FIGURE 2:**
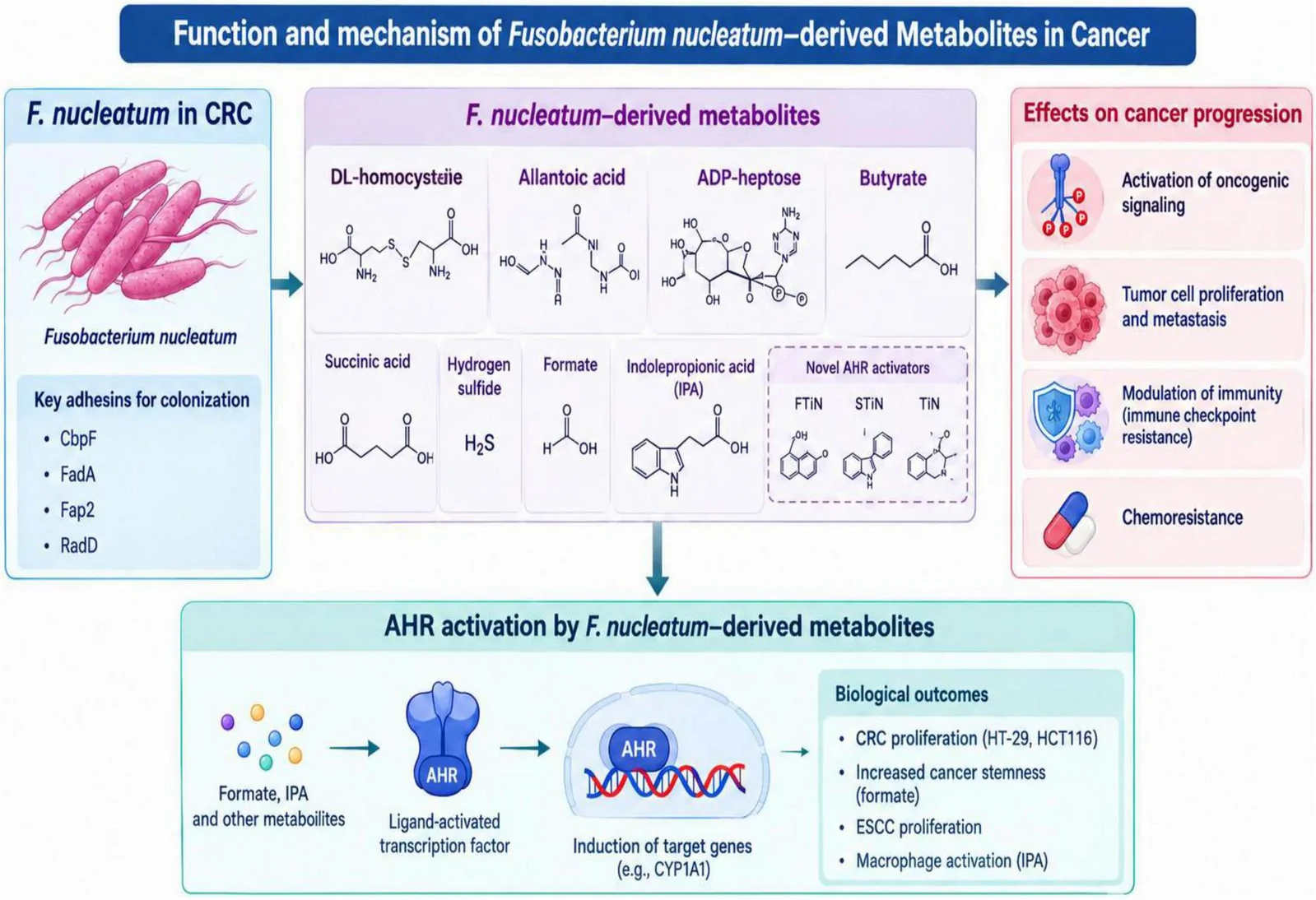
Function and mechanisms of *Fusobacterium nucleatum*-derived metabolites in cancer. *F. nucleatum* is enriched in colorectal cancer (CRC) tissues and produces a diverse range of biologically active metabolites that can influence tumor progression. These include DL-homocystine and allantoic acid, which have been associated with DNA damage; ADP-heptose and butyrate, which cooperatively stimulate NF-κB signaling and promote tumor-cell proliferation and 5-fluorouracil resistance; succinic acid, which contributes to resistance to immune checkpoint blockade (ICB); and hydrogen sulfide (H2S), generated from cysteine metabolism, which promotes inflammatory responses, autophagy, tumor-cell migration, and pathological alterations of the gut microbiome. Formate and several indole-related metabolites have been reported to activate AHR signaling. IPA has been associated with AHR-mediated macrophage reprogramming, whereas Fusotrisindoline (FTIN), streptindole (STIN), and trisindoline (TIN) are recently identified *F. nucleatum*-associated AHR agonists, with FTIN showing strong activity in specific experimental models. The production of these metabolites is dependent on bacterial strain and culture conditions. In FTIN-sensitive CRC cells, AHR activation induces TERT expression and telomerase activity, thereby promoting proliferation, migration, and invasion. The biological consequences of AHR activation are strongly dependent on cellular context and therefore vary among CRC cell types. In addition, *F. nucleatum*-derived formic acid induces GLUT1 expression and glucose uptake, resulting in enhanced glycolytic flux and lactate accumulation. Increased lactate promotes histone lysine lactylation, which is associated with increased CRC-cell proliferation, migration, and invasion, establishing a metabolic–epigenetic *F. nucleatum*–formic acid–GLUT1–lactate axis. Tryptophan metabolism also generates diverse indole derivatives that can regulate AHR/PXR signaling, epithelial barrier function, inflammation, biofilm formation, and tumor-associated immune responses. Importantly, the production and biological effects of these metabolites vary substantially among clinical isolates and depend on metabolite identity, concentration, bacterial metabolic capacity, cellular context, and disease state. Thus, *F. nucleatum*-derived metabolites represent important mediators linking bacterial metabolism with oncogenic signaling, metabolic and epigenetic reprogramming, immune modulation, chemoresistance, and cancer progression.

AHR is a ligand-activated transcription factor able to sense a wide variety of structurally different endogenous and exogenous molecules functioning as AHR agonists (Burbach et al., 1992; Denison and Nagy, 2003). Normally, AHR regulates expression of genes encoding enzymes of xenobiotic metabolism, including CYP1A1, as well as contributes to immune and epithelial cell differentiation and homeostasis (Stockinger et al., 2021). However, *F. nucleatum* has recently emerged as a source of AHR activators important for cancer progression. Besides its involvement in AHR-driven CRC proliferation discussed above, *F. nucleatum* promotes ESCC proliferation via activation of the AHR pathway (Yin et al., 2023). Therefore, there is a possibility that AHR-activating *F. nucleatum* metabolites, including formate and IPA, are part of the interaction between *F. nucleatum* and ESCC. Nevertheless, some fundamental questions remain unanswered. There is no information about the mechanism of formate’s effect on AHR activity, although IPA, suggested to be produced by *F. nucleatum*, has no strong support from genomic evidence, since the corresponding biosynthesis pathway seems to be absent from the *F. nucleatum* genome (Kaur et al., 2019; Ternes et al., 2022). Finally, AHR activation is highly context-dependent and can lead to divergent cellular consequences. For example, the canonical AHR agonist TCDD (2,3,7,8-tetrachlorodibenzo-p-dioxin) promotes proliferation of H508 and SNU-C4 human CRC cell lines, while suppressing proliferation of RKO cells. Thus, it is still unclear how *F. nucleatum* activates AHR and what its role is in CRC progression.

Importantly, Won et al. (2025) in a bioRxiv preprint, identified three AHR-active metabolites associated with *F. nucleatum*: FTIN, STIN, and TIN. Using AHR reporter assays, mRNA induction analysis, cell-based AHR-binding assays, *in silico* modeling, and pharmacological and genetic perturbation of AHR signaling, the authors reported that these metabolites activate AHR under their experimental conditions. Among them, FTIN showed the strongest activity and was associated with AHR/ARNT-dependent induction of CYP1A1 and, in susceptible CRC cell lines, TERT expression and tumor-associated phenotypes. These findings are potentially important but should be interpreted as preprint evidence requiring independent validation and peer-reviewed confirmation, particularly because the biological effects were strongly dependent on the CRC cell line and experimental context. Under Won et al. (2025) experimental culture conditions, FTIN was produced at the highest level, showed the strongest binding affinity to AHR, and was the most potent inducer of canonical AHR signaling, as evidenced by CYP1A1 transcription and activity. Genetic and pharmacological manipulation revealed that FTIN-induced biological activity requires AHR and depends on its genomic, ARNT-dependent, and DNA-binding activity rather than non-genomic signaling. From the functional point of view, FTIN increased proliferation, migration, and invasion in some, but not all, CRC cell lines, with SNU-C4 showing a particularly pronounced response under the experimental conditions examined by Won et al. (2025). In contrast, HCT116 and HT-29 cells did not exhibit the same FTIN-induced phenotypes despite retaining AHR reporter responses. These findings indicate that the biological consequences of FTIN-mediated AHR activation are strongly dependent on the molecular and cellular context of individual CRC cell lines.

Recently, the Fna clades have been shown to vary in pathogenicity in CRC, with clade 2 of Fna (Fna C2) exhibiting higher invasiveness to CRC cells than Fna C1 and being more abundant than Fna C1 and other *F. nucleatum* subspecies in CRC specimens compared with normal adjacent colonic mucosa. Despite the controversy regarding the existence and classification of Fna C2, Won et al. (2025) results reveal that all *F. nucleatum* subspecies tested, including Fna, Fnn, Fnp, and Fnv, are efficient FTIN producers, including Fna C2 strains CTI-01, CTI-05, ATCC 51191, and EVAG_002 (alias 7_1). Thus, the data acquired for the model strain Fnn ATCC 23726 may extend to other FTIN-producing *F. nucleatum* subspecies and constitute a part of the virulence factors of *F. nucleatum*. Activation of AHR by FTIN in CRC cell proliferation *in vitro* and *in vivo* is in line with recent reports highlighting the role of *F. nucleatum*-mediated AHR signaling in cancer development. According to one report, *F. nucleatum*-dependent activation of AHR drives proliferation of esophageal squamous cell carcinoma, but the molecular compound responsible for AHR activation is unidentified. Another study has suggested IPA as an AHR-activating metabolite of *F. nucleatum* capable of stimulating macrophage proliferation and indirectly driving CRC cell proliferation (Song et al., 2025). Importantly, the identification of FTIN, STIN, and TIN by Won et al. (2025) provides a broader view of *F. nucleatum*-associated AHR activation. Under their experimental culture conditions, IPA was not detected at measurable levels, whereas FTIN, STIN, and TIN were produced and activated AHR, with FTIN showing the strongest activity. This difference does not necessarily contradict the IPA-associated effects reported by Song et al. (2025), because bacterial metabolite production can vary according to strain, substrate availability, growth conditions, and culture environment. Thus, IPA should be regarded as a reported and potentially context-dependent *F. nucleatum*-associated AHR ligand rather than as a universal metabolic product of the species. Conversely, FTIN, STIN, and TIN represent distinct AHR-active metabolites identified under specific experimental conditions, with FTIN showing a cell-line-dependent AHR–TERT response. This finding is in agreement with *in silico* analysis revealing that the genome of *F. nucleatum* ATCC 25586 does not encode the pathway for generation of IPA from L-tryptophan. However, production of IPA from alternative metabolites not present in our culture media cannot be excluded.

It has been shown that formate secreted by *F. nucleatum* enhances AHR signaling, promotes Transwell invasion of HCT116 CRC cells, boosts CRC stemness, and participates in CRC metastasis (Ternes et al., 2022). The molecular mechanism mediating the stimulation of AHR signaling by formate has not been revealed yet. Interestingly, FTIN, the most potent AHR agonist discovered in this study, failed to promote Transwell migration of HCT116 cells in experimental conditions (Won et al., 2025). This difference may mean that activation of AHR by FTIN and enhancement of AHR signaling by formate involve different downstream programs in CRC. The question of whether FTIN and formate function independently, synergistically, or in a conditional way will allow us to understand the overall role of *F. nucleatum*-derived metabolites in CRC progression. Furthermore, Won et al. (2025) metabolic and genetic studies allowed identification of indole as the entry point of FTIN/STIN/TIN biosynthesis. Deletion of tnaA led to the loss of indole production and failure of all three metabolites’ production. Consistent with the metabolic phenotype, the tnaA mutant failed to activate AHR and lost the ability to stimulate proliferation, migration, and invasion of SNU-C4 cells. Furthermore, the mutant lost the ability to enhance SNU-C4 tumor growth *in vivo*, while remaining able to grow normally and attach to and invade CRC cells. These data allow proposing small-molecule *TnaA* inhibitors as potential agents for CRC treatment, especially those subtypes that remain responsive to EGFR blockage. In addition, by using gene expression profiling followed by targeted validation, Won et al. (2025) identified TERT as an important downstream target of AHR signaling and a potential factor determining the cell line-specific FTIN response. FTIN increased TERT expression via the AHR/ARNT pathway and elevated telomerase activity. On the other hand, TERT depletion abrogated the proliferative effect of FTIN, while overexpression of TERT stimulated CRC proliferation. Therefore, based on these results, we propose the following model, according to which FTIN activates AHR, which drives a transcriptional program involving TERT, leading to tumor cell growth in FTIN-sensitive CRC subtypes like SNU-C4. The absence of FTIN-induced phenotypes in HCT116 and HT-29 cells, despite preserved AHR reporter responses, demonstrates the great influence of the cell type-specific context on the biological outcomes of AHR activation. Such dependence may be explained by the presence of different oncogenic mutations and transcriptional programs in individual CRC cell lines. Hence, the genetic factors that make CRC cell lines sensitive to FTIN or resistant, as well as the molecular mechanisms connecting AHR activation to TERT induction and further proliferation, require elucidation. TERT is involved in several cancer hallmarks such as enhanced proliferation, invasion and metastasis, and resistance to apoptosis. Importantly, the involvement of TERT in cancer development may occur both through telomerase-dependent and -independent ways.

Indole metabolites can exert highly context-dependent effects through AhR and related host pathways (Bansal et al., 2010). Their biological activity varies according to metabolite identity, concentration, cellular target, and disease context. Thus, rather than assigning a uniform pro- or antitumor effect to AhR activation, the available evidence supports a metabolite- and cell-specific model in which *F. nucleatum*-derived indoles may differentially influence epithelial cells and tumor-associated immune cells (Scano et al., 2025). Biofilm formation is particularly relevant to CRC, in which biofilm-associated microbial communities have been described as a characteristic feature of the disease. More than 85 gram-positive and gram-negative bacterial species can metabolize tryptophan to indole through the activity of the tryptophanase gene tnaA (Lee and Lee, 2010). In addition to their effects on bacterial physiology, indole and its derivatives can activate host AhR and pregnane X receptor (PXR) signaling and thereby influence intestinal homeostasis and potentially modify responses to chemotherapy (Wang G. et al., 2024). Recent evidence has highlighted the potential immunological consequences of *F. nucleatum*-associated indole metabolites. Song et al. (2025) reported IPA as an AHR-active metabolite associated with *F. nucleatum* culture supernatant and demonstrated that IPA reproduced several of the immunosuppressive effects of the bacterial supernatant, including AHR activation, M2-like macrophage polarization, and reduced CD8^+^ T-cell infiltration in experimental CRC models. Using Liquid chromatography-tandem mass spectrometry (LC-MS/MS), the authors identified IPA in the *F. nucleatum* culture supernatant and showed that pharmacological inhibition of AHR attenuated the IPA-associated effects. However, these findings should be interpreted within the experimental context of that study and should not be generalized to all *F. nucleatum* strains (Song et al., 2025). Although increased indole concentration enhances biofilm formation of an oral *F. nucleatum* isolate, the molecular mechanism of regulation of biofilm formation and virulence-related traits by indole and its derivatives in CRC-associated strains of *F. nucleatum* is still unknown. *F. nucleatum* plays an important role in the structure of oral biofilms, where it provides interactions between commensal and pathogenic bacteria, and has been suggested to serve as a source of colon-associated *F. nucleatum*. In the context of CRC, *F. nucleatum* is preferentially enriched in tumors with bacterial biofilms (Scano et al., 2025). Moreover, these *F. nucleatum*-positive biofilms are associated with decreased E-cadherin expression, increased IL-6/Signal transducer and activator of transcription 3 (STAT3) signaling, and increased proliferation of crypt cells in adjacent normal colonic mucosa (Scano et al., 2025). Thus, elucidation of mechanisms regulating *F. nucleatum* biofilm formation is crucial for understanding the role of this bacterium in CRC initiation and progression. Since particular virulence factors of *F. nucleatum* can serve as biomarkers of CRC association, identification of the regulatory mechanisms of expression of virulence determinants is important for understanding the interplay between strain-specific properties of bacteria, their community behavior, and disease progression. Scano et al. (2025) work is the first comparative characterization of indole production in clinical *F. nucleatum* isolates and demonstrates that indole-related metabolic traits can regulate bacterial virulence depending on the disease and environmental contexts via regulation of biofilm formation and virulence factor expression. The intestine contains many chemical signaling molecules that mediate communication between host and microbiota as well as between members of the microbiota and play a key role in maintaining gut homeostasis. Metabolites of tryptophan, including indole, play an especially important role in this signaling circuit since they can enhance intestinal barrier functions and regulate host immune responses (Scano et al., 2025). Besides, they can affect the tumor immune microenvironment. However, their biological effects depend on the particular indole derivative. For example, indole-3-acetic acid (IAA) inhibits anti-tumor immunity in pancreatic cancer by AhR activation in tumor-associated macrophages and consequently reduces CD8+ T-cell activation (Scano et al., 2025). In contrast, IAA or IAA-producing bacteria treatment can alleviate experimental colitis in mice, while deficient AhR signaling and lack of IAA, e.g., due to a high-fat diet, increase intestinal inflammation and disrupt intestinal barrier function (Li et al., 2024). Similar context-dependent biological effects can be demonstrated by other indole derivatives. For instance, indole-3-acetaldehyde (IAAD) inhibits cell proliferation and induces apoptosis at low concentrations, thus having a potential protective effect against CRC development, but at concentrations >25 μmol/L promotes epithelial-mesenchymal transition and invasiveness in CRC cells (Dai et al., 2024). Similarly, indole-3-aldehyde alleviates inflammation in experimental colitis and contributes to the recovery of the intestinal epithelial barrier, in part through AhR activation, which, in addition, can promote IL-22 production. IL-22 can provide protection in certain inflammatory conditions, but also can promote tumor growth in the colon (Scano et al., 2025). Altogether, these data highlight the strong dependence of biological effects of indole metabolites on their identity, concentration, cellular context and disease status. Given the well-established enrichment of *F. nucleatum* within CRC tissues, Scano et al. (2025) therefore investigated whether CRC-associated isolates exhibit metabolic characteristics that could contribute to altered intestinal tryptophan metabolism. Comparative profiling demonstrated that *F. nucleatum* isolates obtained from CRC-associated samples generated significantly greater amounts of total indole than isolates associated with other disease conditions. This observation supports a potential relationship between CRC and enhanced *F. nucleatum*-mediated indole production. Consistent with this interpretation, several indole derivatives–including indole, indole-3-aldehyde, and indole-3-carboxaldehyde–that are enriched in fecal samples from patients with CRC showed positive associations with indole production by CRC-derived isolates. Of particular interest, indole-3-aldehyde, which is elevated in CRC-associated samples, was preferentially produced by three CRC-associated *F. nucleatum* isolates: CC2_6JVN3, SB-CTX47T, and SB-CTX3Tcol3 (Scano et al., 2025). These findings raise the possibility that CRC-associated *F. nucleatum* populations undergo metabolic adaptation toward enhanced indole production, potentially in response to selective pressures or chemical cues within the tumor-associated intestinal environment.

Physiological intestinal indole concentrations have been reported to range from 0.3 to 6.6 mM in fecal samples, while *in vitro* experiments have generally employed concentrations within a comparable range of approximately 0.5–5 mM (Scano et al., 2025). At relatively low concentrations of 0.5–1 mM, indole can alter *E. coli* virulence-related phenotypes, including biofilm formation, transcriptional programs, and stress-adaptation responses. In contrast, concentrations of 4–5 mM can suppress bacterial growth and cell division. Scano et al. (2025) therefore examined whether lower indole concentrations could modify the growth characteristics of *F. nucleatum* without producing a direct antimicrobial effect. Exposure to 0.25–2 mM indole primarily decreased the maximal carrying capacity (OD max) while leaving the inflection time largely unaffected (Scano et al., 2025). Notably, CRC-derived isolates exhibited greater sensitivity than isolates originating from non-CRC disease groups. Nevertheless, the limited impact of 1 mM indole on overall growth across the tested derivatives indicates that the observed responses are more consistent with phenotypic modulation than with overt indole-mediated toxicity.

Biofilm profiling further demonstrated substantial isolate-specific variation in biofilm-forming capacity. The CRC-associated isolate SB-CTX3Tcol3 and the Crohn’s-associated isolate EAVG002-7_1 reached maximal biofilm formation at 36 h, identifying both as particularly strong biofilm-producing strains and suggesting an enhanced capacity for persistent colonization and virulence-associated community formation (Scano et al., 2025). Of note, EAVG002-7_1 belongs to a specific *F. nucleatum* clade previously linked to CRC and has been reported to induce colonic tumor formation in mouse models in which most other isolates exhibit limited or absent colonization (Scano et al., 2025). Interestingly, isolates derived from Crohn’s disease displayed the strongest overall biofilm-forming phenotypes, whereas among the CRC-associated isolates, only CC2_3FMU1 and SB-CTX3Tcol3 demonstrated pronounced biofilm formation. When biofilm production was examined specifically at 36 and 48 h–time points corresponding to the onset of the stationary phase, during which both indole production and biofilm-associated phenotypes are commonly enhanced–a positive association between total indole production and biofilm formation was detected exclusively among CRC-associated isolates (Scano et al., 2025). This disease-specific relationship suggests that CRC-derived *F. nucleatum* strains may possess a distinct metabolic coupling between indole signaling and biofilm development. Such coupling could provide a selective fitness advantage within the metabolically and immunologically complex malignant microenvironment, potentially facilitating persistence, community organization, and expression of virulence-associated traits. Thus, rather than functioning solely as a bacterial growth regulator, indole may represent an environmentally responsive signal that links metabolic adaptation to biofilm-associated virulence in CRC-associated *F. nucleatum*.

Zhou et al. (2026) results reveal an unexplored metabolic link between *F. nucleatum* and the metabolism of CRC cells in which the bacterial formic acid induces glycolytic reprogramming followed by histone lactylation. Specifically, the *F. nucleatum* metabolism leads to the production of formic acid, which causes an increase in GLUT1 expression that is responsible for glucose uptake and the glycolytic process. The higher expression of GLUT1 leads to increased glucose uptake and thus more substrates for glycolysis, causing the accumulation of lactate (Zhou et al., 2026). Given that CRC cells display a high level of glycolysis when compared with normal colorectal epithelial cells, the increase in glycolysis leads to high lactate production. The lactate production has implications for epigenetics since both extracellular and intracellular lactate may cause lysine lactylation of histone proteins (Li et al., 2022; Zong et al., 2024). The relationship between lactate metabolism and the regulation of the chromatin structure provides another aspect to understand how the Warburg effect expands from energy metabolism to tumor-associated cellular processes and even tumor immunity. Furthermore, Zhou et al. (2026) found that histone lactylation in CRC cells is correlated with the lactate concentration, since increased lactate concentration correlates with increased histone lactylation, which in turn causes increased proliferation, migration, and invasion of tumor cells. More importantly, using clinical tumor specimens, they provided, to the best of our knowledge, the first evidence showing that the amount of *F. nucleatum* inside the tumors is associated with histone lactylation in CRC cells. In this way, Zhou et al. (2026) study broadens the known role of *F. nucleatum* in colorectal carcinogenesis, which includes promotion of colorectal tumor development through tumor-cell proliferation, suppression of antitumor immunity, induction of local inflammation, and cellular DNA damage. Their study adds one more metabolic–epigenetic activity of the previously characterized bacteria by suggesting that the metabolite of *F. nucleatum* is able to regulate glycolysis and its epigenetics in tumor cells. Zhou et al. (2026) reported an association between *F. nucleatum* exposure, formate-related metabolic changes, GLUT1 expression, lactate accumulation, and histone lactylation in CRC cells. Their experiments showed that formate was associated with increased GLUT1 expression and glucose uptake, accompanied by enhanced glycolytic activity and lactate accumulation. Increased lactate levels were also associated with increased histone lactylation, while histone lactylation was associated with enhanced proliferation, migration, and invasion of CRC cells. Analyses of clinical tumor specimens further showed an association between intratumoral *F. nucleatum* abundance and histone lactylation (Zhou et al., 2026). These findings support a potential metabolic–epigenetic relationship between *F. nucleatum* and CRC-cell behavior. However, the individual interventions used in the study do not necessarily demonstrate that histone lactylation is the unique or indispensable mediator of the observed phenotypes. In particular, pharmacological or metabolic inhibition of upstream pathways can affect multiple cellular processes simultaneously and therefore should not be interpreted as specific evidence that histone lactylation alone mediates the downstream effects. Accordingly, the proposed *F. nucleatum*–formate–GLUT1–lactate–lactylation pathway should be regarded as a supported mechanistic model with experimentally demonstrated links at several individual steps, rather than as a completely established linear causal chain.

*Fusobacterium nucleatum* usually inhabits the oral cavity and metabolizes cysteine (Cys), leading to the formation of hydrogen sulfide (H2S) (Wang et al., 2023). Interestingly, *F. nucleatum* is also regularly detected in stool specimens from patients with CRC. This raises the possibility that metabolic products of this bacterium are involved in CRC-induced intestinal dysbiosis. In Wang et al. (2023) study, the concentration of H2S capable of inducing CRC cell proliferation and migration was identified first and then compared to the concentrations of H2S found in the colonic milieu of patients with CRC. Such an approach allowed estimation of appropriate concentrations of Cys for future experiments. Their results showed that *F. nucleatum* induced inflammation and activation of autophagy in CRC cells and mice (Wang et al., 2023). Moreover, the introduction of Cys, which increased H2S production, intensified autophagy even more. This means that H2S is likely to be one of the mediators of metabolic communication between *F. nucleatum* and CRC cells. Besides its effects on cancer cells, *F. nucleatum* and H2S resulted in significant changes in the composition of the gut microbiome in mice, because *F. nucleatum* and its H2S metabolite induced enrichment of pathogenic bacteria and reduction of beneficial bacteria (Wang et al., 2023). Gene expression analysis of HCT116 cells was performed to find out what genes were affected by *F. nucleatum* and H2S. TNF was identified among genes that were upregulated in both HCT116 + *F. nucleatum* and HCT116 + *F. nucleatum* + Cys groups (Wang et al., 2023). TNF is known to be responsible for the induction of AOM/DSS-induced carcinogenesis in mice and also correlates with bacterial abundance. Moreover, the gene encoding MAP1LC3A, the major autophagy protein, was also changed upon treatment of HCT116 cells with *F. nucleatum* and Cys, supporting Wang et al. (2023) finding about the stimulation of autophagy. Administration of *F. nucleatum* and Cys increased the expression of several genes, which can be involved in tumor progression. For instance, expression of SELE was found to be increased in many metastatic cancers such as colorectal, breast, and prostate cancers compared to normal controls (Smirnov et al., 2006). In addition, CCL20 was found to promote proliferation and migration of CRC epithelial cells (Nandi et al., 2021). CXCL11 is also highly expressed in colon adenocarcinoma and rectal adenocarcinoma and, therefore, could serve as a marker for colon adenocarcinoma (Cao et al., 2021). It is worth noting that the effects of *F. nucleatum* and H2S were not limited to the signaling pathways in tumor cells (Wang et al., 2023). *F. nucleatum* and its H2S metabolite caused significant changes in the composition of the intestinal microbiome, which manifested in enrichment of pathogenic bacteria and depletion of probiotics. All the above results suggest that *F. nucleatum* metabolism involves the production of pathogenic H2S, which increases inflammatory responses and autophagy in CRC cells and promotes the development of a pathological gut microbiome (Wang et al., 2023). Taking into account the pro-tumor effect of H2S, inhibition of its production or signaling by pharmacological means could represent a promising therapeutic strategy for the prevention of *F. nucleatum*-associated CRC progression. However, the therapeutic value of such an approach needs further investigation since it is necessary to clarify whether the inhibition of *F. nucleatum*-derived H2S affects only tumor-promoting pathways or also interferes with other microbial and host metabolic processes.

### 4.2 Periodontitis

*Fusobacterium nucleatum* has been commonly isolated from subgingival dental plaque in both healthy and periodontally compromised individuals, which indicates a possible role of this microorganism in maintenance of periodontal homeostasis as well as disease progression and the associated dysbiotic microbial communities (Moore and Moore, 1994; Brennan and Garrett, 2019). In contrast to most saccharolytic bacteria, *F. nucleatum* uses peptides and amino acids as a nutrient source and produces butyrate and ammonia through fermentative processes mainly based on amino acids such as glutamate and lysine (Sakanaka et al., 2022). Importantly, metatranscriptomics studies have shown that although the relative abundance of *F. nucleatum* in healthy and periodontitis-associated microbial communities is similar, the metabolic activity of this bacterium is highly different under these two conditions (Jorth et al., 2014). It is important to note that *F. nucleatum* has been found not only in association with periodontal disease but also with several systemic pathological disorders, including adverse pregnancy outcomes and CRC (Brennan and Garrett, 2019). Therefore, elucidation of the impact of metabolic plasticity of *F. nucleatum* and interactions with other members of microbial communities on its biological role is essential for understanding its contribution to health and diseases.

Recently, a previously unknown metabolic interaction between *Streptococcus gordonii* and *F. nucleatum* has been described (Sakanaka et al., 2015). In the course of the interaction, *S. gordonii* metabolizes arginine through the arginine deiminase system (ADS), where arginine is converted to ornithine with simultaneous generation of ammonia and ATP (Sakanaka et al., 2022). Ornithine is exported by the ArcD arginine–ornithine antiporter of *Streptococcus gordonii* and becomes available for *F. nucleatum* (Sakanaka et al., 2022). Metabolic interaction leads to stimulation of the growth and biofilm formation of *F. nucleatum*. However, the impact of ornithine consumption on the metabolic physiology of *F. nucleatum* and downstream effects of such cross-feeding interaction for periodontal disease are not fully understood yet. To investigate the above questions, Sakanaka et al. (2022) aimed to further characterize the metabolic interactions mediated by *F. nucleatum* in oral multispecies microbial communities and to test the possibility that such interactions could influence the pathogenic potential of microbial consortia. The data obtained demonstrate a novel ecological role of *F. nucleatum* as a metabolic mediator promoting metabolite exchanges between early and late oral colonizers and establishing metabolic conditions favorable for expansion and dissemination of Porphyromonas gingivalis (Sakanaka et al., 2022). These findings further support the idea that there is a wide range of amino acid cross-feeding interactions occurring in supragingival and subgingival microbial communities and may serve as a metabolic mechanism of dysbiosis in periodontal disease. An important observation made in Sakanaka et al. (2022) study is the metabolic cooperation between *S. gordonii* and *F. nucleatum*, where arginine-derived ornithine is further converted to putrescine. In the trophic network, the product of the metabolic interaction between two microorganisms alters the biofilm-associated phenotype of *P. gingivalis*. Metabolically, *F. nucleatum* converts ornithine to putrescine through ornithine decarboxylation, which consumes cytoplasmic protons and generates proton motive force and hence increases acid resistance as well as the energy gain for *F. nucleatum* (Barbieri et al., 2019). At the same time, the removal of ornithine by *F. nucleatum* stimulates continued functioning of the ADS in *S. gordonii* and supports its energy metabolism and ATP synthesis. From the evolutionary and ecological points of view, the interaction represents a case where metabolic complementarity contributes to the stabilization of multispecies microbial communities. The combined activity of the *S. gordonii* ADS and the ornithine decarboxylase pathway of *F. nucleatum* results in the creation of a mutually beneficial metabolic loop, where a product synthesized by one microorganism is efficiently consumed by another. Such metabolic cross-feeding interaction decreases competition for limiting nutrients and provides physiological benefit to both organisms. As a result, the cooperative metabolic configuration could be evolutionarily selected for since it ensures efficient utilization of resources and improves the fitness of the involved microorganisms in nutrient-limited oral biofilms.

## 5 Context-dependent effects of *F. nucleatum*-derived metabolites on immunotherapy

The effects of *F. nucleatum* on immunotherapy are highly context-dependent and cannot be inferred from bacterial abundance alone. In particular, apparently opposing effects on anti-PD-1 efficacy have been reported depending on the anatomical localization of *F. nucleatum*, the metabolite involved, the tumor model and molecular subtype, the immune compartment affected, and the route and context of immunotherapy. Evidence from intestinal/systemic *F. nucleatum* and circulating succinate has linked this axis to impaired anti-PD-1 responses through suppression of cGAS–IFN-β signaling and reduced CD8^+^ T-cell trafficking. In the study by Jiang et al. (2023) increased intestinal *F. nucleatum* and circulating succinate were associated with reduced sensitivity to anti-PD-1 therapy rather than enhanced immunotherapy efficacy. Depletion of *F. nucleatum* with metronidazole reduced circulating succinate and restored tumor sensitivity to anti-PD-1 treatment, supporting a detrimental role for the intestinal *F. nucleatum*–succinate axis in this experimental context. Importantly, these findings should not be generalized to all *F. nucleatum*-associated immunotherapy responses, because intratumoral *F. nucleatum* and its locally produced metabolites may exert different effects depending on bacterial localization, metabolite identity, tumor subtype, and immune context. Although it has been previously suggested that *Fusobacterium* metabolites affect the efficacy of ICB (Figure 3), their identity and contribution to ICB efficacy have remained elusive. Jiang et al. (2023) identified *F. nucleatum*-associated succinate as a metabolite linked to impaired ICB responsiveness in CRC. In this experimental context, the relevant metabolic signal was associated with intestinal/systemic exposure and circulating succinate, which suppressed cGAS–IFN-β signaling and impaired CD8+ T-cell trafficking, thereby reducing the efficacy of anti-PD-1 therapy. Thus, this finding should be interpreted specifically as evidence for a *F. nucleatum*–succinate axis operating within an intestinal/systemic context rather than as evidence that *F. nucleatum* abundance per se universally predicts resistance to immunotherapy. Importantly, the effect of *F. nucleatum* on immunotherapy is not uniformly detrimental. In contrast to the intestinal/systemic succinate-associated phenotype described above, intratumoral *F. nucleatum* has been reported to enhance anti-PD-1 responsiveness in MSS CRC models. In this setting, locally produced butyrate inhibited Histone Deacetylase (HDAC) 3/8 activity, promoted *Tbx21* activation, and reduced CD8+ T-cell exhaustion, thereby improving anti-tumor immune responses. The difference between these findings highlights the importance of anatomical localization and metabolite identity: intestinal/systemic *F. nucleatum*-associated succinate and intratumoral *F. nucleatum*-derived butyrate represent distinct exposure compartments and molecular mechanisms and should not be treated as interchangeable evidence. It has been reported previously that *F. nucleatum* can affect immunosuppression of the tumor microenvironment (Kostic et al., 2013; Serna et al., 2020). Indeed, the Fap2 protein of *F. nucleatum* binds the inhibitory receptor TIGIT on tumor-infiltrating lymphocytes and induces inhibitory signaling, thereby suppressing effector-cell functions and weakening antitumor immune responses (Gur et al., 2015; Xu et al., 2021). Moreover, *F. nucleatum* can promote macrophage infiltration via CCL20 activation and induce M2 polarization of macrophages to facilitate tumor metastasis (Xu et al., 2021). Taken together, these data suggest that *Fusobacterium* can create a favorable environment for immunosuppression within CRC. Despite all the above, the relationship between *Fusobacterium* and the efficacy of immunotherapy seems to be context-dependent. Gao et al. (2021) have demonstrated that intratumoral administration of *F. nucleatum* can improve the efficacy of PD-L1 blocking therapy in CRC. However, Jiang et al. (2023) results show that intestinal colonization with *F. nucleatum* reduced the sensitivity of CRC to anti-PD-1 monoclonal antibody therapy via a mechanism dependent, at least partially, on its metabolite succinic acid. Indeed, when the *frdA* gene of *F. nucleatum* was disrupted, and succinate production was consequently impaired, the bacterium was unable to reduce the sensitivity of CRC tumors to anti-PD-1 therapy and suppress the cGAS pathway. Based on these results, it is possible to hypothesize that the ICB-repressing properties of intestinal *Fusobacterium* are not only due to its presence but also due to the metabolic activity and ability to produce succinic acid. The differences observed between the results can be attributed to the differences in the experimental setup and biological consequences of bacterial localization. In the study of Gao et al. (2021), intratumoral administration of *F. nucleatum* provided the bacterium with direct contact with the tumor tissue and increased the efficacy of PD-L1 blockade, which coincided with activation of NF-κB (p65) and increased PD-L1 expression in tumor cells. Therefore, the observed effect depends mainly on the direct contact of the bacterium with the tumor. As previously suggested, intratumoral administration of specific bacterial strains can increase antigen presentation and interaction with dendritic cells, which increases the efficacy of antitumor immunity (Wang et al., 2022). Some bacterial strains can possess an oncolytic or tumor-remodeling activity and increase PD-L1 expression in tumor cells, thus potentially transforming immunologically “cold” tumors into “hot” ones, susceptible to ICB-based treatment (Lin et al., 2021). Thus, it is obvious that the influence of *F. nucleatum* on ICB cannot be considered without taking into account its localization and interaction with the host immune system.

**FIGURE 3:**
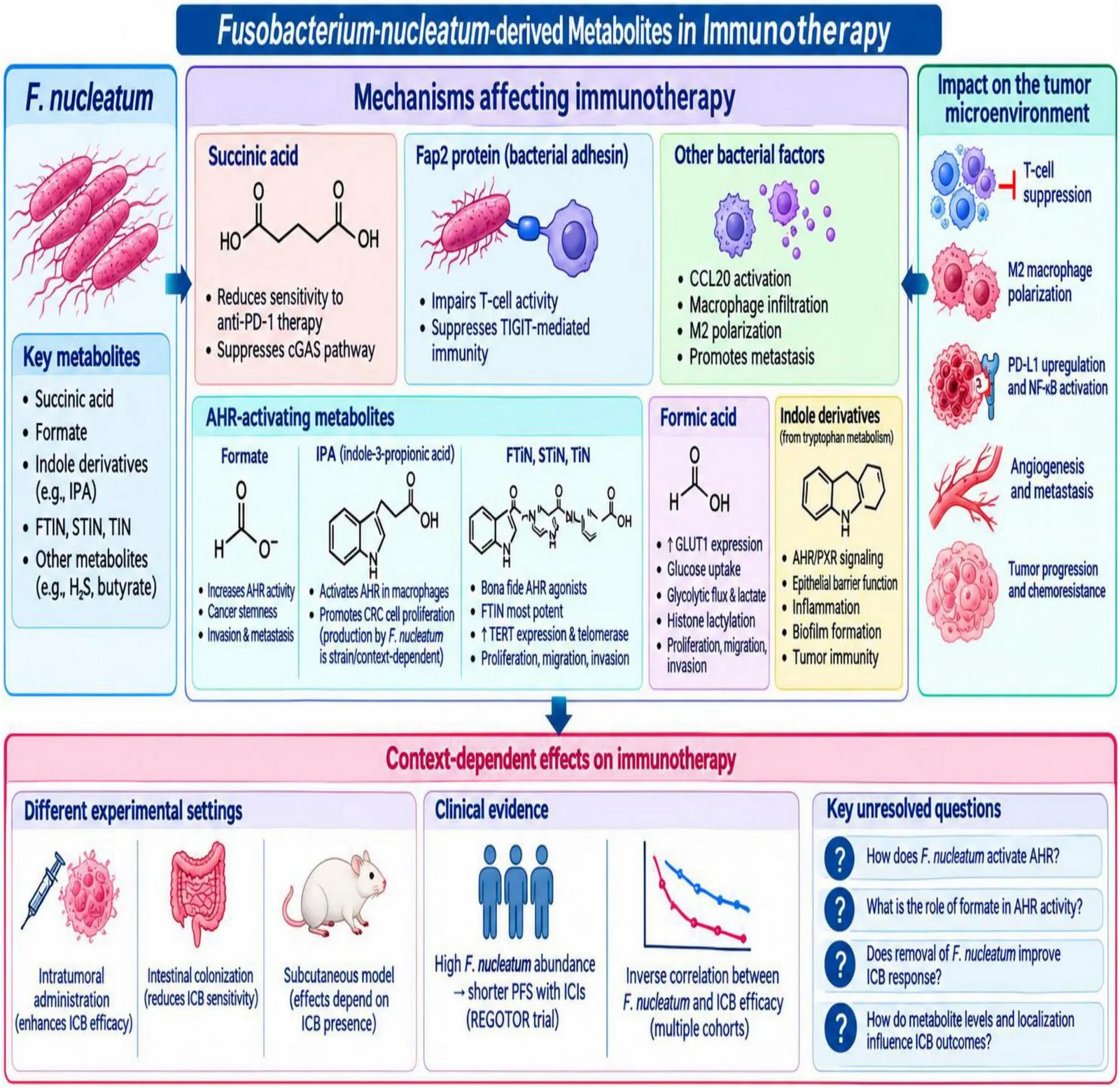
Context-dependent effects of *Fusobacterium nucleatum*-associated metabolites on immunotherapy. *F. nucleatum* can influence colorectal cancer (CRC) responses to immune checkpoint blockade (ICB) through mechanisms that differ according to bacterial localization, metabolite production, tumor molecular phenotype, immune context, and treatment setting. In intestinal colonization models, *F. nucleatum*-associated succinate increases circulating succinate and suppresses cGAS–IFN-β signaling, thereby reducing antitumor immune activation and CD8^+^ T-cell trafficking and decreasing sensitivity to anti-PD-1 therapy. Disruption of the *frdA* gene, which reduces succinate production, prevents this phenotype in the experimental model, supporting a causal role for the *F. nucleatum*–succinate axis. In contrast, in specific MSS CRC models, locally available *F. nucleatum*-associated butyrate has been linked to enhanced anti-PD-1 responses through inhibition of HDAC3/8, increased *Tbx21* transcription, reduced PD-1 expression, and restoration of CD8^+^ T-cell effector function. Butyrate has also been reported to reduce *MLH1* expression and promote dMMR/MSI-associated changes, potentially increasing responsiveness to PD-1/PD-L1 blockade. These findings should not be interpreted as evidence that butyrate universally counteracts the immunosuppressive effects of *F. nucleatum*. Rather, they illustrate that the immunological consequences of *F. nucleatum* are strongly dependent on bacterial strain, administration route, colonization site, tumor model, immune and molecular status, metabolite concentration and compartment, and ICB treatment. Fecal, culture-supernatant, circulating, and tumor concentrations represent distinct exposure compartments and should not be assumed to reflect equivalent physiological exposure.

Clinical data also indicate an unfavorable correlation between the abundance of *Fusobacterium* and ICB. The relationship between *F. nucleatum* abundance and immunotherapy outcome is context-dependent and should be interpreted according to bacterial localization, tissue compartment, metabolite profile, CRC subtype, immune state, and treatment setting (Wang F. et al., 2021). Importantly, an association between microbial abundance and clinical outcome should not be interpreted as evidence of treatment-specific predictive value unless the measurement has been prospectively validated for differential treatment benefit. Jiang et al. (2023) results provide additional support for the hypothesis of a possible relationship between the abundance of *Fusobacterium* within the intestinal or tumor microbiota and ICB resistance. Furthermore, according to experimental results, tumor-stimulating effects of *F. nucleatum* in subcutaneous tumor models critically depend on the presence of ICB therapy. Monocolonization with *F. nucleatum* without anti-PD-1 mAb treatment did not accelerate tumor progression and did not suppress antitumor immunity. These results are consistent with previous work about bacteria whose effects appear only in the context of ICB therapy. In addition, the underlying mechanism revealed that intestinal *F. nucleatum* did not accumulate in heterotopic tumors in germ-free (GF) mice that received *F. nucleatum* orally and anti-PD-1 mAb intraperitoneally. It is crucial to note that this result shows that the presence of *F. nucleatum* in the tumor itself is not needed for ICB resistance. Intestinal *F. nucleatum* can affect systemic immune and metabolic pathways through circulating microbial metabolites, succinic acid being one of them (Jiang et al., 2023). Jiang et al. (2023) showed that metronidazole-induced depletion of intestinal *F. nucleatum* restored anti-PD-1 mAb efficacy *in vivo*. However, erythromycin did not have this effect. Although these findings provide preclinical proof-of-concept that intervention targeting intestinal *F. nucleatum* can modulate ICB resistance, the broad-spectrum activity of metronidazole should be taken into account because changes in the broader microbial community may contribute to the observed effect.

The translational implications of these findings should nevertheless be interpreted cautiously. Although fecal *F. nucleatum* abundance and circulating succinate were associated with immunotherapy outcomes in the reported cohort, these observations should currently be considered exploratory and hypothesis-generating rather than evidence of clinically validated predictive biomarkers. In particular, fecal *F. nucleatum* abundance and circulating succinate may provide complementary information about the intestinal microbial and metabolic state, but their ability to predict benefit from ICB requires validation in prospective clinical cohorts. Differences in bacterial strain, detection methodology, biological compartment, and metabolite measurement may also influence the observed associations. Accordingly, prospective studies are required to establish reproducible treatment-specific predictive value and clinically relevant thresholds for the *F. nucleatum*–succinate axis (Jiang et al., 2023). Apart from the development of biomarkers, therapeutic approaches that reduce intestinal *F. nucleatum* and succinic acid production and signaling can potentially increase the efficiency of anti-PD-1 treatment. However, prospective clinical studies are needed to establish predictive value and clinically relevant thresholds and to investigate whether it is possible to manipulate the *F. nucleatum*–succinate axis in order to improve immunotherapy. Recent literature further supports the view that *F. nucleatum* represents a multifaceted component of CRC biology, with potential implications for immune modulation, biomarker development, and therapeutic response. In addition to metabolite-dependent mechanisms, *F. nucleatum* can influence the tumor immune microenvironment through bacterial virulence factors and interactions with immune-cell signaling pathways. These observations place the metabolite-centered mechanisms discussed above within a broader translational framework and suggest that the clinical significance of *F. nucleatum* may depend on the integration of bacterial abundance, localization, metabolic phenotype, host immune status, and treatment context (Incognito et al., 2025).

In Wang X. et al. (2024) study, they showed a paradoxical role of *F. nucleatum* in MSS CRC, a molecular subclass of CRCs generally known for low immunotherapy response rates to immune checkpoint inhibitors. While *F. nucleatum* colonization itself has been demonstrated to accelerate tumor progression, they discovered that its presence increases the efficacy of anti-PD-1 treatment in MSS CRC through an immune-mediated mechanism. Mechanistically, this increase in the effectiveness of anti-PD-1 therapy results from a direct influence of *F. nucleatum* on T-bet gene expression through production of butyric acid. In turn, this gene activation leads to downregulation of PD-1 in CD8^+^ TILs, which enhances their functional potential and increases the number of CD8^+^ TILs. As a consequence of this process, *F. nucleatum* colonization contributes to the improvement of antitumor immunity in combination with anti-PD-1 therapy. Additionally, Wang X. et al. (2024) showed that the possibility exists that *F. nucleatum* abundance or its butyrate-producing phenotype could help identify MSS CRC tumors that are more responsive to anti-PD-1 therapy. However, this potential biomarker application remains preliminary and requires validation in prospective clinical cohorts. By performing experiments in humanized mouse models of MSS CRC, Wang X. et al. (2024) found that *F. nucleatum* colonization results in two apparently opposite effects. First, without the use of immune checkpoint inhibitors, *F. nucleatum* increases the rate of tumor growth. At the same time, when anti-PD-1 therapy is used, *F. nucleatum* colonization transforms this poorly responding tumor model into a responding tumor and even induces tumor suppression. The immunological studies revealed that the mechanism underlying this phenomenon is connected with reduction of PD-1 in CD8^+^ TILs and subsequent restoration of their cytotoxic potential in the intratumoral microenvironment. Therefore, Wang X. et al. (2024) show that, depending on particular immunological and therapeutic background, *F. nucleatum* can be an organism promoting reactivation of antitumor immunity. This result is especially important in view of MSS CRC being a dominant type of CRC, which does not generally show an effective response to anti-PD-1 therapy. Wang X. et al. (2024) also identified the main metabolite secreted by *F. nucleatum*, which leads to changes in TIME and increases the efficiency of anti-PD-1 therapy. Using the T-cell/CRC organoid co-culture system, current study found the *F. nucleatum*-conditioned medium (CM) decreased CD8^+^ T-cell exhaustion, manifested in a reduction of PD-1 expression and an increase in the frequencies of IFN-γ^+^, TNF-α^+^, and GZMB^+^ CD8^+^ T cells. The conclusion about the critical role of butyric acid was confirmed by experiments involving an *F. nucleatum* butyric acid biosynthesis-deficient mutant, which did not demonstrate the influence of wild-type *F. nucleatum* on CD8^+^ T cells. In turn, the addition of butyric acid reproduced the immunomodulating effects of *F. nucleatum*. Consistently with the *in vitro* findings, both *F. nucleatum* and butyric acid, but not the butyric-acid-deficient *F. nucleatum* mutant, induced antitumor immunity in humanized and syngeneic mice. Collectively, these findings indicate that the effect of *F. nucleatum* on ICB is not uniform but depends on bacterial localization, metabolic output, tumor subtype, and therapeutic context. In particular, succinate-associated signaling may contribute to ICB resistance, whereas butyrate production has been linked to enhanced anti-PD-1 responses in specific MSS CRC models. The interpretation of these findings is consistent with previous data that showed that butyric acid can induce antitumor immunity (Kang et al., 2023; Zhu et al., 2023). Namely, butyric acid was shown to increase the effector function of CD8^+^ T cells in cancer through mechanisms related to ID2, TLR5, and T cell receptor signaling (Kang et al., 2023; Zhu et al., 2023). Wang X. et al. (2024) extend this finding by identifying the effect of butyric acid on immune regulation, in particular, its ability to attenuate CD8^+^ T-cell exhaustion via repression of PD-1 expression. Since butyric acid has a relatively short systemic half-life due to fast degradation and excretion, it is clear that local presence of butyric acid is also essential for its immunomodulatory properties. Therefore, Wang X. et al. (2024) findings indicate that immunological properties of butyric acid produced by *F. nucleatum* may depend not only on systemic levels but also on the location of its synthesis within the intestinal and tumor-associated microenvironment. At the molecular level, they discovered the epigenetic mechanism of butyric acid action on PD-1 repression in CD8^+^ TILs (Wang X. et al., 2024). Butyric acid inhibits the activity of histone deacetylases HDAC3 and HDAC8, causing increased H3K27ac enrichment at the *Tbx21* promoter region. Increased acetylation causes higher *TBX21* expression, and this transcription factor acts as a transcriptional repressor of PD-1 and other inhibitory T-cell receptors (Wang X. et al., 2024). Thus, *F. nucleatum*-butyric acid axis is linked to the epigenetic regulation of T-cell transcription, making CD8^+^ TILs cytotoxic again.

Importantly, the role of *F. nucleatum* in antitumor immunity cannot be considered only as antitumor. *In situ* analysis of human CRC tissue revealed the formation of *F. nucleatum*-rich tumor niches that are depleted in CD3^+^ T cells but enriched in neutrophils and macrophages involved in inflammation (Galeano Niño et al., 2022). These data clearly show the context dependence of *F. nucleatum* biology and indicate that *F. nucleatum* effects are dependent on bacterial localization, metabolite synthesis, immune cell composition, and therapeutic treatment. Importantly, these findings do not establish that the immunostimulatory effects of butyrate generally outweigh the immunosuppressive effects associated with *F. nucleatum*. Rather, they demonstrate that, under the specific MSS CRC and anti-PD-1 treatment conditions examined, the *F. nucleatum*–butyrate–HDAC3/8–TBX21 pathway can contribute to restoration of CD8^+^ T-cell effector function and improved antitumor responses. The relative contribution of this pathway compared with other immunosuppressive mechanisms of *F. nucleatum* remains to be determined in comparative experimental studies. Overall, Wang X. et al. (2024) establish the *F. nucleatum*-butyric acid-HDAC3/8-TBX21 axis as a novel mechanism enhancing the efficacy of anti-PD-1 therapy in MSS CRC. Through HDAC3/8 inhibition, butyric acid increases the H3K27ac enrichment at the *Tbx21* promoter, increases *TBX21* expression, and leads to PD-1 down-regulation in CD8^+^ TILs. Thus, the epigenetic remodeling of T-cell transcription reactivates the cytotoxicity of anti-tumor immunity in the context of anti-PD-1 therapy. Besides discovering the previously unknown pro-immunomodulatory role of *F. nucleatum* in immunotherapy-resistant MSS CRC, the data therefore raise the hypothesis that *F. nucleatum* abundance may be associated with anti-PD-1 responsiveness in MSS CRC; however, prospective clinical validation is required before such measurements can be considered predictive biomarkers for treatment selection. These findings provide a rationale for investigating whether modulation of local butyrate exposure or its downstream epigenetic effects could influence anti-PD-1 responses; however, therapeutic efficacy and clinical feasibility remain to be established.

Additionally, Ding et al. (2025) provided evidence for a potentially immunotherapy-sensitizing effect of *F. nucleatum*-associated butyrate in CRC. Their study showed that *F. nucleatum* inhibited *MLH1* expression through activation of the autophagy–lysosomal pathway, resulting in mismatch-repair deficiency (dMMR) and microsatellite instability (MSI), which are associated with increased responsiveness to ICB. Importantly, *ex vivo* and *in vivo* experiments demonstrated that butyrate suppressed MLH1 protein expression at low concentrations, while the combination of butyrate with PD-L1 inhibition increased the efficacy of immunotherapy. However, this potentially beneficial effect should be interpreted in the context of the experimental models used and should not be extrapolated to *F. nucleatum* as a whole, given that other *F. nucleatum*-associated metabolites, particularly succinate, have been linked to impaired anti-PD-1 responses (Ding et al., 2025). Importantly, it was determined that *F. nucleatum* is an independent risk factor for MLH1 deficiency, suggesting that this microorganism might play a role in the regulation of MLH1. Ding et al. (2025) provided evidence that *F. nucleatum* and its metabolite butyrate can reduce MLH1 protein expression in CRC models. In CRC tissues, higher *F. nucleatum* abundance was also associated with MSI and MLH1 deficiency; however, these tissue-level observations are associative and do not by themselves establish a direct causal relationship between bacterial abundance and MSI. In cell and tissue models, *F. nucleatum* exposure and butyrate treatment reduced MLH1 protein levels, whereas *MLH1* mRNA levels were not substantially altered under the tested conditions, supporting a post-transcriptional mechanism. RNA-sequencing analysis further indicated enrichment of lysosomal pathways, and pharmacological inhibition of the autophagy–lysosome pathway partially restored MLH1 protein levels (Ding et al., 2025). Thus, the experimental evidence supports a model in which *F. nucleatum*-associated butyrate can promote MLH1 protein loss through an autophagy–lysosome-associated mechanism. However, *MLH1* depletion should not be considered synonymous with functionally demonstrated dMMR or newly acquired MSI unless these phenotypes are directly measured. Similarly, the enhanced tumor response observed with combined butyrate and PD-L1 blockade in a subcutaneous mouse model demonstrates an experimental treatment effect, but does not by itself establish that conversion from MSS/pMMR to MSI/dMMR is the sole or necessary mediator of this response. Further studies directly measuring MMR function and MSI during bacterial or metabolite exposure are required to determine whether an MSS-to-MSI transition occurs and whether it accounts mechanistically for the observed immunotherapy sensitization. These results provide another strategy for the enhancement of immunotherapy sensitivity by modulating *MLH1* expression through *F. nucleatum*. *MLH1* promoter methylation is the major molecular mechanism for MLH1 deficiency in sporadic MSI CRC (Boland and Goel, 2010). In previous research, the connection of *F. nucleatum* not only with MSI but also with *MLH1* promoter methylation and DNMT activity was shown (Xia et al., 2020). However, in the experiments, neither *MLH1* promoter methylation nor DNMT activity changed in CRC cell lines and CRC tissues under the stimulation of *F. nucleatum* or its metabolites. This result argues against *MLH1* promoter methylation as the main cause of MLH1 protein deficiency. Increasing evidence shows that MLH1 expression can be controlled by other regulatory mechanisms, such as post-translational ones. The study of Chen et al. (2004) demonstrated the hydrolysis of MLH1 protein via Caspase-3 under the effect of tumor necrosis factor-related apoptosis-inducing ligands or the topoisomerase II inhibitor etoposide. Similarly, the research of Daniels has shown that the tyrosine kinase inhibitors imatinib and nilotinib lead to MLH1 protein depletion without changing *MLH1* mRNA levels. Subsequently, it has been demonstrated that MLH1 protein is degraded via the lysosomal pathway (Daniels et al., 2022). To investigate whether *F. nucleatum* regulates MLH1 through a post-translational mechanism, Ding et al. (2025) performed RNA sequencing in COLO cells exposed to *F. nucleatum* supernatant and observed enrichment of lysosome-related pathways. In addition, pharmacological inhibition of the autophagy–lysosome pathway partially attenuated the reduction in MLH1 protein induced by *F. nucleatum* supernatant or butyrate (Ding et al., 2025). These experiments therefore implicate lysosomal/autophagy-related processes in the regulation of MLH1 protein abundance. However, pathway enrichment or changes in autophagy-associated markers should not be interpreted as direct evidence of increased autophagic flux unless flux is specifically measured. Thus, the available findings support involvement of the autophagy–lysosome system in *F. nucleatum*-associated MLH1 loss, while the precise contribution of autophagic flux and lysosomal degradation remains to be defined.

Previously, it has been demonstrated that the conversion of MSS/pMMR tumors to MSI/dMMR through the inactivation of the *MMR* gene MLH1 leads to tumor regression in mice with immune system integrity by changing the immune microenvironment (Germano et al., 2017). Thus, taking into account the ability of *F. nucleatum* to induce MLH1 deficiency, the target therapy for *F. nucleatum* or its components could be a potential strategy for the sensitization of immunotherapy. However, the transplantation of the overall microbiota would be very risky because of the carcinogenic properties of *F. nucleatum*. Thus, the selective targeting of the components of *F. nucleatum* responsible for the induction of dMMR could be a more reasonable approach. Previously, studies have shown that SCFAs, the main metabolites of *F. nucleatum*, have a regulatory impact on the immune system and are related to the reactions to immunotherapy (Nomura et al., 2020; Brennan et al., 2021). Thus, Ding et al. (2025) decided to examine the link between SCFAs and the MSI/dMMR phenotype. The *ex vivo* and *in vivo* experiments have shown that butyrate, an SCFA, can suppress MLH1 protein expression at low concentrations. Moreover, *in vivo* experiments have revealed that the combination of butyrate and PD-L1 inhibitors increases the efficiency of immunotherapy. This result expands the current knowledge about the mechanisms of the influence of microbial metabolites on immunotherapy responses and provides experimental proof of the potential of converting pMMR/MSS CRC into an immunotherapy-responsive phenotype. Although the combination of ICIs and butyrate demonstrates therapeutic potential, there are some limitations that should be considered carefully. Firstly, neither *ex vivo* models nor subcutaneous tumor models reproduce the complexity of the native tumor microenvironment. Therefore, the translational applicability and feasibility of the combination of butyrate and ICIs need to be verified in more physiologically relevant experimental models. Secondly, the toxicity of butyrate needs to be comprehensively studied, especially from the standpoint of dose dependence and systemic tolerance. Despite these limitations, it has been demonstrated in the present study that *F. nucleatum* inhibits *MLH1* expression via butyric acid, leading to the mechanism of MSI formation and a potential avenue of MSS-to-MSI conversion. This finding also provides a mechanistic explanation of how gut microbiota-derived metabolites could increase the efficacy of immunotherapy. Overall, current evidence indicates that the immunological consequences of *F. nucleatum*-derived metabolites are determined by the anatomical compartment, metabolite produced, tumor context, and immune state. Intestinal/systemic *F. nucleatum*-associated succinate may impair anti-PD-1 efficacy by limiting cGAS–IFN-β signaling and CD8+ T-cell trafficking, whereas intratumoral *F. nucleatum*-derived butyrate may enhance anti-PD-1 responses in MSS CRC through HDAC3/8 inhibition, *Tbx21* activation, and reduced CD8+ T-cell exhaustion. Accordingly, *F. nucleatum* abundance should not be considered an independent predictor of immunotherapy benefit or resistance without specifying the tissue compartment, metabolite, tumor subtype, immune context, and therapeutic setting. More broadly, current measurements of *F. nucleatum* abundance or metabolite levels should be regarded as candidates for biomarker development rather than clinically validated predictive biomarkers. Prospective studies using standardized, compartment-specific assays and predefined thresholds are required to determine whether these measurements provide prognostic information, predict benefit from a specific treatment, or both.

## 6 Conclusion

Increasing evidence shows that *F. nucleatum* participates in disease development via mechanisms that go beyond merely colonization and traditional virulence factors. Metabolic activity of the microorganism provides a crucial interface between the microbe, host, and the local microbiota. By metabolizing, *F. nucleatum* may produce or modify bioactive metabolites, including SCFAs, indole derivatives, succinate, hydrogen sulfide, and formate, that may affect the processes of inflammatory cell recruitment, immune cell differentiation, epithelial barrier integrity, tumor cell metabolism, epigenetics, biofilm development, and therapy response. Hence, the pathogenicity of *Fusobacterium* is determined not only by bacterial abundance but also by metabolites produced by bacteria within a certain host niche. While SCFAs are known to mediate neutrophil recruitment and inflammatory responses in periodontal disease, butyrate has antitumor and immunotherapy-enhancing activity under certain CRC conditions. In addition, the effects of indole derivatives on AhR-dependent pathways may be either protective or tumor-promoting depending on metabolite nature, its concentration, target cell, and the disease condition. Also, conflicting results about the effects of *F. nucleatum* on ICB demonstrate that *Fusobacterium* effects greatly depend on the bacterial localization and metabolic context: succinate from *F. nucleatum* in the intestinal tract is linked with suppression of anti-PD-1 response, while other experimental models show that butyrate from *F. nucleatum* increases antitumor immunity via activation of CD8+ T-cells. Currently, there are some challenges ahead before these concepts can be implemented in clinical practice. First, most of the mechanistic evidence is based on the use of *in vitro* and animal models, while human studies often rely on associations rather than causation. Second, metabolite concentrations used in experimental settings may differ from metabolite concentrations in a certain anatomical niche in patients. Furthermore, diet, host genetics, microbiota composition, drug exposure, disease stage, bacterial strain, and localization may affect metabolite production and activity. An important consideration for future studies is the integrated influence of dietary composition, host genetic background, and microbial community structure on microbial metabolite production and host responsiveness. Dietary substrates can alter the metabolic capacity and relative abundance of microbial populations, whereas host genetic and physiological differences may affect metabolite sensing, transport, metabolism, and downstream signaling. Thus, inter-individual variation in diet, host factors, and microbial composition may contribute to differences in the abundance, localization, and biological effects of *F. nucleatum*-associated metabolites. A nutrigenomics framework may therefore be useful for understanding this variability and for designing future studies that integrate dietary, host-genetic, microbiome, and metabolomic information. Importantly, this framework represents a general diet–microbiome–host principle and does not by itself establish a specific *F. nucleatum* metabolic pathway.

Consequently, prospective studies in humans are needed to clarify whether certain *Fusobacterium*-derived metabolites or metabolic signatures can be used as reliable biomarkers of disease progression or therapy response. Overall, current data provide support for a transition of the paradigm from “*Fusobacterium* as a pathogen” to “*Fusobacterium* as a metabolically adaptable host microenvironment regulator”. Metabolites produced by *Fusobacterium* may serve as chemical mediators connecting microbial ecology with inflammation, immune regulation, tissue metabolism, tumor development, and therapeutic responsiveness. Identification of the metabolite-centered mechanisms of *Fusobacterium* involvement in disease progression with strain, spatial, and quantitative resolution may result in the development of more precise interventions that will disrupt pathogenic microbial functions and preserve host-microbiota relationships.
